# Two-phase rejective sampling and its asymptotic properties

**DOI:** 10.1093/jrsssb/qkaf002

**Published:** 2025-02-10

**Authors:** Shu Yang, Peng Ding

**Affiliations:** Department of Statistics, North Carolina State University, Raleigh, NC 27695, USA; Department of Statistics, University of California, Berkeley, CA 94720, USA

**Keywords:** covariate adjustment, design property, double sampling, multi-phase sampling

## Abstract

Rejective sampling improves design and estimation efficiency of single-phase sampling when auxiliary information in a finite population is available. When such auxiliary information is unavailable, we propose to use two-phase rejective sampling (TPRS), which involves measuring auxiliary variables for the sample of units in the first phase, followed by the implementation of rejective sampling for the outcome in the second phase. We explore the asymptotic design properties of double expansion and regression estimators under TPRS. We show that TPRS enhances the efficiency of the double-expansion estimator, rendering it comparable to a regression estimator. We further refine the design to accommodate varying importance of covariates and extend it to multi-phase sampling. We start with the theory for the population mean and then extend the theory to parameters defined by general estimating equations. Our asymptotic results for TPRS immediately cover the existing single-phase rejective sampling, under which the asymptotic theory has not been fully established.

## 1 Introduction

Two-phase sampling, also known as double sampling, is a cost-effective method in large surveys, initially using auxiliary variables for broad measurement in the first phase, followed by targeted measurement of primary study variables in the second phase. Pioneered by Neyman (1938) and further developed by Cochran (2007) and Legg and Fuller (2009), two-phase sampling integrates auxiliary variables in both the design and analysis stages. In the design stage, stratified sampling is often used to leverage discrete auxiliary variables from the first phase to inform the selection of the second-phase sample. This strategy enhances the efficiency of estimators like weighted expansion estimators (Oh & Scheuren, 1983) over simple random sampling of the same size. In the analysis stage, auxiliary variables can be used to enhance the efficiency of estimation, for example, through regression adjustment. Regression estimators work for both discrete and continuous variables, providing gains in efficiency over traditional approaches without auxiliary variables.

Regression estimators, however, have the practical drawback of having potential negative weights. Methods such as rejective sampling (Hájek, 1964; Hájek & Dupač, 1981) and balanced sampling (Deville & Tillé, 2004, 2005; Tillé & Favre, 2005; Valliant et al., 2000; Yates, 1946) have been developed to mitigate the problem of negative weights associated with regression estimators. Rejective sampling involves selecting a sample through a basic sampling design, which is then rejected if the difference between the sample mean and the population mean of an auxiliary vector exceeds a specified threshold. Extensive analyses of such samples are provided by Hájek (1964) and Hájek and Dupač (1981). Herson (1976) extends this by describing a rejection procedure where the rejection region is defined as a cube, and introduces the concept of a balanced sample—later referred to as partially balanced or restricted samples by others. Balanced sampling ensures that the sample mean closely matches the population mean of auxiliary variables, retaining the optimality of the ratio estimator under many polynomial regression models (Royall & Herson, 1973). Valliant et al. (2000) further explore the use of balanced or partially balanced samples with model-based estimators. Deville and Tillé (2004) introduce the cube method for creating such balanced samples, even with unequal probabilities and multiple auxiliary variables. They also establish that a conditional Poisson design with appropriate inclusion probabilities can be used to create a balanced sample (Deville & Tillé, 2005). Fuller (2009b) demonstrates that the mean and variance of the regression estimator are asymptotically equivalent for both rejective and original samples, and that the variance estimator for the original sample is appropriate for the rejective sample. Empirical comparisons of the cube method and rejective sampling are provided by Legg and Yu (2010). Fuller et al. (2017) introduce a bootstrap variance estimation method for single-phase rejective sampling. Yang et al. (2023) combine rejective sampling and rerandomization in experiments to improve both external and internal validity. However, most of the existing design methods are limited to single-phase sampling and require auxiliary variable data for the entire population.

We introduce two-phase rejective sampling (TPRS) and explore its asymptotic design properties with commonly used estimators, namely weighted expansion and regression estimators. In design-based inference, the characteristics of finite populations are fixed, and the randomness arises from the sampling process. Two-phase rejective sampling allows for using both continuous and discrete auxiliary variables in the design stage, relaxing the requirement of observing auxiliary variables in the whole finite population typically associated with single-phase sampling. Two-phase rejective sampling offers several practical benefits: it ensures an unbiased sample of the target population, reduces the variance of the population mean estimator for covariates, prevents the selection of samples with extreme auxiliary variable values, and reduces the likelihood of negative weights in regression estimators. Additionally, TPRS enhances the efficiency of double-expansion estimators across multiple outcomes to match the performance of regression estimators, without the need of multiple model fitting for outcomes. We present the first derivation of the asymptotic distribution result under TPRS, complementing Fuller (2009b)’s work, which established the consistency and asymptotic variance of the regression estimator without deriving the asymptotic distribution under single-phase rejective sampling. Furthermore, we refine the TPRS design to account for varying importance of covariates and extend it to multi-phase sampling. Under TPRS, we also discuss general parameter estimation, including population proportion, variance, and quantiles, beyond the population mean of the study variable.

We focus on rejective sampling and regression adjustment for using general auxiliary variables in two-phase sampling, but other design and analysis strategies are also viable. Cube methods (Deville & Tillé, 2004) offer a design strategy extending to two-phase settings, ensuring first-order inclusion probabilities for an unbiased sample although not controlling auxiliary variable total discrepancies between phases. Best-Choice Rerandomization (Wang & Li, 2023) offers a design strategy for rejective sampling by repeatedly sampling and selecting the sample with the best covariate balance. For other example of analysis strategies, calibration weighting, proposed by Estevao and Särndal (2010) for two-phase sampling, aligns with the two-phase regression estimator when using generalized least squares distance. Deville and Särndal (1992) and Deville et al. (1993) demonstrate that calibration estimators are asymptotically equivalent across various distance metrics. Based on this, we conjecture that calibration estimators in two-phase sampling, with or without rejective sampling, share similar limiting distributions. Also, calibration estimators complement regression adjustments by ensuring positive or bounded weights (Deville & Tillé, 2004). Due to the complexity of deriving an analytic expression for joint inclusion probabilities, Deville and Tillé (2005) propose a general approximation of variance estimation based on the residual technique. We have applied similar techniques, allowing for soft calibration instead of hard calibration, and accommodating multi-phase sampling and general parameter estimation.

The paper proceeds as follows. Section 2 provides a review of existing design and analysis strategies of using auxiliary variables. Sections 3 and 4 discuss TPRS with simple random sampling and general sampling, respectively. Section 5 provides several extensions. Section 6 reports simulation results and an application that illustrates the finite-sample performance of TPRS. We relegate all technical details and proofs to the online supplementary material.

## 2 A review of design and analysis strategies of using auxiliary variables

Consider a finite population with a known size *N*. For each unit *i*, $x_{i}$ is a *p*-dimensional auxiliary variable, and $y_{i}$ is the study variable of interest. We focus on a scalar *y*, but our theory extends to vector *y* immediately. The finite population quantities $F_{N}=\{(x_{1},y_{1}),(x_{2},y_{2}),\ldots ,(x_{N},y_{N})\}$ are fixed. For simplicity, we suppress the subscript *N* on F when there is no ambiguity. The parameter of interest is the finite population mean of the study variable ${\bar{y}}_{0}=N^{-1}\sum_{i=1}^{N}y_{i}$.

Two-phase sampling offers an efficient and economical method for conducting large-scale surveys. In this section, we review the existing methods for using auxiliary variables in two-phase sampling to enhance estimation efficiency and identify areas needing new strategies.

### 2.1 Existing design strategy: two-phase stratified sampling

During the design stage, auxiliary variables are incorporated by selecting a second-phase sample through stratified sampling. Strata are based on first-phase variables, either directly from discrete variable categories or via discretization of continuous variables. We define $x_{i}=(x_{1i},\ldots ,x_{Hi})$ as the stratum indicator vector, where $x_{hi}=1$ if unit *i* is in stratum *h*, and $x_{hi}=0$ otherwise. Two-phase stratified sampling proceeds as follows:

Step 1. From the population F, select a first-phase sample A of size $n_{I}$. For i∈A, record $x_{i}$. Define the sample size in each stratum *h* as $m_{h}$ for h=1,…,H. The total first-phase sample size is $n_{I}=\sum_{h=1}^{H}m_{h}$.

Step 2. From each stratum *h*, randomly select $r_{h}$ units, independently across strata, as the second-phase sample B. For i∈B, record the study variable $y_{i}$. The total second-phase sample size is $n_{\mathrm{II}}=\sum_{h=1}^{H}r_{h}$.

In two-phase sampling, *the double-expansion estimator* ($\pi^{*}$ estimator; Särndal et al., 2003) and *the reweighted expansion estimator* (REE; Kott & Stukel, 1997; Oh & Scheuren, 1983) are the canonical estimators for the population mean. The $\pi^{*}$ estimator,


$${\hat{y}}_{\pi^{*}}=\frac{1}{N}\sum_{i\in B}\frac{y_{i}}{\pi_{\mathrm{II}i}^{*}},$$

where $\pi_{\mathrm{II}i}^{*}=\pi_{Ii}\pi_{\mathrm{II}i|A}$ with $\pi_{Ii}=P(i\in A)$ and $\pi_{\mathrm{II}i|A}=P(i\in B|i\in A)$, adjusts individual observations by the product of their inclusion probabilities in both phases. We can also replace *N* in ${\hat{y}}_{\pi^{*}}$ by $\sum_{i\in B}(\pi_{\mathrm{II}i}^{*}{)}^{-1}$, emulating the Hajek estimator. However, the combined probability $\pi_{\mathrm{II}i}^{*}=\pi_{Ii}\pi_{\mathrm{II}i|A}$ is generally not the unconditional probability of a unit being in the phase-II sample $P(i\in B)=\sum_{A}\pi_{Ii}\pi_{\mathrm{II}i|A}P(A)$, the average probability across all possible first-phase samples, unless $\pi_{\mathrm{II}i|A}$ is invariant of the first-phase sample (Beaumont & Haziza, 2016; Fuller, 2009a). The REE,


$${\hat{y}}_{\mathrm{REE}}=\frac{1}{N}\sum_{h=1}^{H}\left(\sum_{i\in A}\frac{x_{hi}}{\pi_{Ii}}\right)\frac{\sum_{i\in B}(\pi_{\mathrm{II}i}^{*}{)}^{-1}x_{hi}y_{i}}{\sum_{i\in B}(\pi_{\mathrm{II}i}^{*}{)}^{-1}x_{hi}},$$

recalculates the mean estimate for each stratum by a modified ratio estimator, where the stratum-specific mean of *x* is approximated based on phase-I data, while the coefficient is derived from phase-II data. These estimators are often more efficient than the sample mean estimator under simple random sampling with the same size $n_{\mathrm{II}}$.

### 2.2 Existing analysis strategy: regression adjustment

Using auxiliary variables in the analysis phase of two-phase sampling can improve estimation efficiency. These variables can be discrete, continuous, or a combination. The REE is one strategy for utilizing discrete auxiliary variables. More broadly, regression adjustment allows for utilizing general auxiliary variables, not limited to discrete types.

In simple random sampling across both phases, we observe variable *x* in phase I and variables (x,y) in phase II. Then, we can calculate the mean estimates ${\bar{x}}_{I}=n_{I}^{-1}\sum_{i\in A}x_{i}$ from phase I and $\left({\bar{x}}_{\mathrm{II}},{\bar{y}}_{\mathrm{II}})=n_{\mathrm{II}}^{-1}\sum_{i\in B}(x_{i},y_{i}\right)$ from phase II. The two-phase regression estimator is


$${\bar{y}}_{\mathrm{reg}}={\bar{y}}_{\mathrm{II}}-({\bar{x}}_{\mathrm{II}}-{\bar{x}}_{I}{)}^{T}{\hat{\beta }}_{\mathrm{II}},$$

where


(1) $${\hat{\beta }}_{\mathrm{II}}={\left\{\sum_{i\in B}\left(x_{i}-{\bar{x}}_{\mathrm{II}}\right){\left(x_{i}-{\bar{x}}_{\mathrm{II}}\right)}^{T}\right\}}^{-1}\sum_{i\in B}\left(x_{i}-{\bar{x}}_{\mathrm{II}}\right)\left(y_{i}-{\bar{y}}_{\mathrm{II}}\right)$$

is the regression coefficient based on the phase-II sample.

The regression estimator ${\bar{y}}_{\mathrm{reg}}$ can also be written as a weighted average of the second-phase outcomes, where the corresponding weights are called the generalized regression estimation weights. The regression estimator provides improved efficiency over ${\bar{y}}_{\mathrm{II}}$ in large samples if $x_{i}$ is predictive of $y_{i}$. However, issues like large numbers of *x*-variables or imbalance between ${\bar{x}}_{\mathrm{II}}$ and ${\bar{x}}_{I}$ can lead to extreme or negative weights and affect the performance of ${\bar{y}}_{\mathrm{reg}}$ in finite samples. Researchers have explored various methods to mitigate the issue of negative weights associated with regression estimators, such as balanced sampling or rejective sampling, but these approaches have mainly been studied in single-phase sampling (Deville & Tillé, 2004; Fuller, 2009b; Tillé & Favre, 2005; Valliant et al., 2000; Yates, 1946).

We have reviewed existing design and analysis strategies in multi-phase sampling for better efficiency, but currently it lacks strategies for integrating both continuous and discrete variables in the design stage. We will explore rejective sampling in multi-phase sampling to fill the gap.

## 3 Two-phase rejective sampling

### 3.1 Setup and notation

Throughout this paper, we use *u* and *v* to be generic notation for variables, which can be components of either *x* or *y*. Define E(⋅∣F), cov(⋅∣F), and var(⋅∣F) as expectation, covariance, and variance under the sampling design. The finite population mean for $u_{i}$ and covariance for $u_{i}$ and $v_{i}$ are


(2) $${\bar{u}}_{0}=\frac{1}{N}\sum_{i=1}^{N}u_{i},\;V_{uv,0}=\frac{1}{N-1}\sum_{i=1}^{N}(u_{i}-{\bar{u}}_{0})(v_{i}-{\bar{v}}_{0}{)}^{T}.$$

The phase-I sample mean for $u_{i}$ and covariance for $u_{i}$ and $v_{i}$ are


(3) $${\bar{u}}_{I}=\frac{1}{n_{I}}\sum_{i\in A}u_{i},\;V_{uv,I}=\frac{1}{n_{I}-1}\sum_{i\in A}(u_{i}-{\bar{u}}_{I})(v_{i}-{\bar{v}}_{I}{)}^{T}.$$

We assume $V_{xx,0}$ and $V_{xx,I}$ are positive definite.

We introduce general notation to be used throughout the paper. For a generic vector *v*, let $v^{⊗2}$ denote $vv^{T}$. Define $O_{P}(1)$ as the random variable bounded in probability, and $o_{P}(1)$ as the random variable that converges to zero in probability as *N* increases. Let $z_{\alpha }$ represent the 100αth quantile of the standard normal distribution.

### 3.2 Two-phase rejective sampling with simple random sampling

Rejective sampling aims for balanced phase-II sample selection by comparing the mean differences between phase-II and phase-I samples. We define TPRS with simple random sampling as follows.

Definition 1 **TPRS with simple random sampling**

Two-phase rejective sampling with simple random sampling consists of two steps:

Step 1. Select a phase-I sample A of size $n_{I}$ by simple random sampling. For i∈A, record $x_{i}$.

Step 2. Select a phase-II sample B of size $n_{\mathrm{II}}\leq n_{I}$ by simple random sampling from phase-I sample A. Accept the phase-II sample if

$$Q_{I}=({\bar{x}}_{\mathrm{II}}-{\bar{x}}_{I}{)}^{T}{\left\{(n_{\mathrm{II}}^{-1}-n_{I}^{-1})V_{xx,I}\right\}}^{-1}({\bar{x}}_{\mathrm{II}}-{\bar{x}}_{I})<\gamma^{2},$$

where $\gamma^{2}>0$ is a prespecified constant, and $(n_{\mathrm{II}}^{-1}-n_{I}^{-1})V_{xx,I}$ is the phase-II design variance of ${\bar{x}}_{\mathrm{II}}-{\bar{x}}_{I}$ given A. For i∈B, record $y_{i}$.

In Definition 1, if $Q_{I}$ is below a specified threshold $\gamma^{2}$, the phase-II sample is considered balanced and accepted for further analysis. Based on the TPRS, the sample mean estimator for the population mean ${\bar{y}}_{0}$ is


$${\bar{y}}_{\mathrm{II}}=n_{\mathrm{II}}^{-1}\sum_{i\in B}y_{i}.$$

### 3.3 Large-sample design properties

We investigate the asymptotic design-based property of ${\bar{y}}_{\mathrm{II}}$ under TPRS in Definition 1. For asymptotic inference, we adopt the framework of Isaki and Fuller (1982), which establishes the asymptotic properties of estimators within a fixed sequence of populations and corresponding random samples. This involves a series of nested finite populations $\left(F_{N_{1}}\subset F_{N_{2}}\subset F_{N_{3}}\subset \cdots \right)$ and sequences of samples with increasing sample sizes $(A_{n_{I,1}}\subset A_{n_{I,2}}\subset A_{n_{I,3}}\subset \cdots$ and $B_{n_{\mathrm{II},1}}\subset B_{n_{\mathrm{II},2}}\subset B_{n_{\mathrm{II},3}}\subset \cdots$). For simplicity, we will not explicitly mention the dependence of $N_{t}$, $n_{I,t}$, and $n_{\mathrm{II},t}$ on *t*, and refer to the asymptotic regime as the scenario where index *t* or *N* goes to infinity.

The linear projection of $y_{i}$ onto $x_{i}$ in the finite population F is ${\bar{y}}_{0}+(x_{i}-{\bar{x}}_{0}{)}^{T}\beta_{0}$, where


$$\beta_{0}=\arg\;\min_{\beta }\sum_{i=1}^{N}{\left\{y_{i}-{\bar{y}}_{0}-{\left(x_{i}-{\bar{x}}_{0}\right)}^{T}\beta \right\}}^{2},$$

which equals


(4) $$\beta_{0}={\left\{\sum_{i=1}^{N}(x_{i}-{\bar{x}}_{0}{)}^{⊗2}\right\}}^{-1}\sum_{i=1}^{N}(x_{i}-{\bar{x}}_{0})(y_{i}-{\bar{y}}_{0})=V_{xx,0}^{-1}V_{xy,0}.$$

Assumption 1 Assume F contains IID samples from a superpopulation of (x,y) with two conditions: (i) The sequence of F has finite (4+δ) moments for some δ>0, implying $E(|y{|}^{4+\delta })<\infty$ and $E(\|x{\|}^{4+\delta })<\infty$ with respect to the superpopulation model; (ii) $\lim_{N\to \infty }n_{I}/N=f_{I,0}$ and $\lim_{N\to \infty }n_{\mathrm{II}}/n_{I}=f_{\mathrm{II},I}$ for some $0\leq f_{I,0}\leq 1$ and $0\leq f_{\mathrm{II},I}\leq 1$.

Assumption 1(i) sets moment conditions for the superpopulation, which aids in applying central limit theorems. Chen and Rao (2007) studied sufficient moment conditions on the finite population that ensure the asymptotic normality of estimators in two-phase sampling. Assumption 1(i) implies that for any components *u* and *v* of *x* and *y*,


(5) $$\lim_{N\to \infty }V_{uv,I}=\lim_{N\to \infty }V_{uv,0}=V_{uv}\;a.s.,$$

where $V_{uv}$ is a constant vector or matrix. Assumption 1(ii) defines the sampling fractions $f_{I,0}$ and $f_{\mathrm{II},I}$ for the phase-I and phase-II samples, respectively. Sampling from a finite population or phase-I sample without replacement can introduce dependency among the individual samples. The sampling fractions serve to adjust for such dependency when calculating the asymptotic design variances and covariances.

Define the adjusted outcome as $e_{i}=y_{i}-x_{i}^{T}\beta_{0}$. The error in the sample mean estimator ${\bar{y}}_{\mathrm{II}}$ of the population mean ${\bar{y}}_{0}$ decomposes into three parts:


(6) $$\begin{array}{rl} n_{\mathrm{II}}^{1/2}({\bar{y}}_{\mathrm{II}}-{\bar{y}}_{0}) & =n_{\mathrm{II}}^{1/2}({\bar{x}}_{\mathrm{II}}-{\bar{x}}_{I}{)}^{T}\beta_{0}+n_{\mathrm{II}}^{1/2}\{{\bar{y}}_{\mathrm{II}}-{\bar{y}}_{I}-({\bar{x}}_{\mathrm{II}}-{\bar{x}}_{I}{)}^{T}\beta_{0}\}+n_{\mathrm{II}}^{1/2}({\bar{y}}_{I}-{\bar{y}}_{0})\\ & \equiv T_{1}+T_{2}+T_{3}. \end{array}$$

In (6), $T_{1}=n_{\mathrm{II}}^{1/2}({\bar{x}}_{\mathrm{II}}-{\bar{x}}_{I}{)}^{T}\beta_{0}$ and $T_{2}=n_{\mathrm{II}}^{1/2}({\bar{e}}_{\mathrm{II}}-{\bar{e}}_{I})$ represent the errors of *x* and *e* in the phase-II sample conditional on the phase-I sample, respectively, and $T_{3}=n_{\mathrm{II}}^{1/2}({\bar{y}}_{I}-{\bar{y}}_{0})$ represents the error of *y* in the phase-I sample. The limiting distribution of $\left(T_{1},T_{2},T_{3}\right)$ is given in the following lemma.

Lemma 1 Suppose Assumption 1 holds. Without the rejection step in TPRS in Definition 1, $\left(T_{1},T_{2},T_{3}\right)$ in the decomposition (6) has the following limiting distribution:

$$\;\left(\begin{array}{c} T_{1}\\ T_{2}\\ T_{3} \end{array}\right)|F\to N\left\{\left(\begin{array}{c} 0\\ 0\\ 0 \end{array}\right),\left(\begin{array}{ccc} \left(1-f_{\mathrm{II},I}\right)V_{yx}V_{xx}^{-1}V_{xy}, & 0 & 0\\ 0 & \left(1-f_{\mathrm{II},I}\right)V_{ee} & 0\\ 0 & 0 & f_{\mathrm{II},I}\left(1-f_{I,0}\right)V_{yy} \end{array}\right)\right\},$$

a.s. for all sequences of finite populations, where $V_{uv}$ is defined in (5), and $f_{\mathrm{II},I}$ and $f_{I,0}$ are defined in Assumption 1.

With the rejection step in TPRS, the distribution of the error $n_{\mathrm{II}}^{1/2}({\bar{y}}_{\mathrm{II}}-{\bar{y}}_{0})$ equals the conditional distribution of $n_{\mathrm{II}}^{1/2}({\bar{y}}_{\mathrm{II}}-{\bar{y}}_{0})|(Q_{I}<\gamma^{2})$ without rejective sampling. We use the normalized distance $D_{I}=\{(n_{\mathrm{II}}^{-1}-n_{I}^{-1})V_{xx,I}{\}}^{-1/2}({\bar{x}}_{\mathrm{II}}-{\bar{x}}_{I})$ for the phase-II sample to represent the acceptance criteria $Q_{I}<\gamma^{2}$ by $D_{I}^{T}D_{I}<\gamma^{2}$. By Lemma 1, $D_{I}\to N(0,I_{\;p})$ and thus $D_{I}^{T}D_{I}\to \chi_{\;p}^{2}$ a.s. We define the superpopulation squared correlation between *x* and *y* as $R^{2}=\{\mathrm{corr}(x,y){\}}^{2}=V_{yx}V_{xx}^{-1}V_{xy}/V_{yy}.$ We show the limiting distribution of $n_{\mathrm{II}}^{1/2}({\bar{y}}_{\mathrm{II}}-{\bar{y}}_{0})$ comprises three independent random components.

Theorem 1 Suppose Assumption 1 holds. Under TPRS in Definition 1, ${\bar{y}}_{\mathrm{II}}$ follows the limiting distribution:

(7) $$\begin{array}{rl} n_{\mathrm{II}}^{1/2}({\bar{y}}_{\mathrm{II}}-{\bar{y}}_{0})|\left(Q_{I}<\gamma^{2}\right)\to & {\left\{(1-f_{\mathrm{II},I})V_{yy}R^{2}\right\}}^{1/2}L_{\;p,\gamma^{2}}\\ & +{\left\{(1-f_{\mathrm{II},I})V_{yy}(1-R^{2})\right\}}^{1/2}Z_{1}\\ & +{\left\{\;f_{\mathrm{II},I}\left(1-f_{I,0}\right)V_{yy}\right\}}^{1/2}Z_{2}, \end{array}$$

where

(8) $$L_{\;p,\gamma^{2}}∼\chi_{\;p,\gamma^{2}}S\Gamma_{\;p}^{1/2},$$

with $\chi_{\;p,\gamma^{2}}∼\chi_{\;p}|(\chi_{\;p}^{2}\leq \gamma^{2})$, S follows a uniform distribution on {−1,1}, $\Gamma_{\;p}∼$  Beta{1/2,(p−1)/2}, and $S\Gamma_{\;p}^{1/2}$ is the first coordinate of the uniform random vector over the (p−1)-dimensional unit sphere, $Z_{1}$ and $Z_{2}$ are standard normal variables, and $\left(L_{\;p,\gamma^{2}},Z_{1},Z_{2}\right)$ are jointly independent.

The random variable $L_{\;p,\gamma^{2}}$, first introduced in Li et al. (2018) for rerandomization in causal inference, is also relevant here. Our context is more complex due to the uncertainty in phase-I estimators.

Denote $v_{\;p,\gamma^{2}}=\mathrm{var}(L_{\;p,\gamma^{2}})$, which equals $v_{\;p,\gamma^{2}}=P(\chi_{\;p+2}^{2}\leq \gamma^{2})/P(\chi_{\;p}^{2}\leq \gamma^{2})$ by (8) and is less than or equal to 1 (Li et al., 2018; Morgan & Rubin, 2012).

Corollary 1 Under Assumption 1 and TPRS in Definition 1, the asymptotic design variance of $n_{\mathrm{II}}^{1/2}({\bar{y}}_{\mathrm{II}}-{\bar{y}}_{0})$ is

$$\left[(1-f_{\mathrm{II},I})\left\{1-\left(1-v_{\;p,\gamma^{2}}\right)R^{2}\right\}+f_{\mathrm{II},I}(1-f_{I,0})\right]V_{yy}.$$

The percentage reduction in asymptotic design variance compared with the standard two-phase simple random sampling is

(9) $$\frac{1-f_{\mathrm{II},I}}{1-f_{\mathrm{II},I}f_{I,0}}\left(1-v_{p,\gamma^{2}}\right)R^{2},$$

provided $f_{\mathrm{II},I}f_{I,0}\neq 1$. If $f_{\mathrm{II},I}f_{I,0}=1,$ indicating a census situation, this reduction is zero. Moreover, ${\bar{y}}_{\mathrm{II}}$ exhibits a narrower quantile range under TPRS in Definition 1 than under the standard two-phase simple random sampling.

Without rejective sampling, $\{(1-f_{\mathrm{II},I})V_{yy}R^{2}{\}}^{1/2}L_{\;p,\gamma^{2}}$ in the distribution of (7) with $\gamma^{2}=\infty$ equals $\{(1-f_{\mathrm{II},I})V_{yy}R^{2}{\}}^{1/2}Z_{0}$, where $Z_{0}$, $Z_{1}$, and $Z_{2}$ are independent standard normal variables. When conditioned on $Q_{I}<\gamma^{2}$, the distribution of $L_{\;p,\gamma^{2}}$ is more concentrated around zero than the distribution of $Z_{0}$. This leads to a reduction in variance and quantile range for ${\bar{y}}_{\mathrm{II}}$ following rejective sampling.

From Theorem 1 and Corollary 1, we discuss the trade-off between variance and utility of the sample when selecting $\gamma^{2}$. A lower $\gamma^{2}$ reduces the asymptotic variance of ${\bar{y}}_{\mathrm{II}}$, and in particular, $\{(1-f_{\mathrm{II},I})V_{yy}R^{2}{\}}^{1/2}L_{\;p,\gamma^{2}}$ can be eliminated from the asymptotic distribution (7) if *γ* approaches 0 (Wang & Li, 2022). In the limit with γ→0, the asymptotic distribution of ${\bar{y}}_{\mathrm{II}}$ under TPRS is approximately the same as the sum of two normal random variables $\{(1-f_{\mathrm{II},I})V_{yy}(1-R^{2}){\}}^{1/2}Z_{1}+\{f_{\mathrm{II},I}(1-f_{I,0})V_{yy}{\}}^{1/2}Z_{2}$. However, it also lowers the acceptance rate for the phase-II sample and potentially limits randomness and observations in the tail of the *x* distribution, as discussed by Legg and Yu (2010). This reduces the utility of the sample for unplanned domain analysis, particularly those concerning aspects associated with the tail of the distribution of *x*. In practice, if the primary focus is the population parameter associated with $y_{i}$, we recommend setting $\gamma^{2}$ to the 0.001 quantile of the $\chi_{\;p}^{2}$ distribution. This results in an approximate acceptance rate of 0.001, as in a related experimental design context (Li et al., 2018).

Moreover, when $R^{2}=0$, indicating no correlation between auxiliary and study variables, the rejective procedure does not affect the limiting distribution for ${\bar{y}}_{\mathrm{II}}$. Conversely, with $R^{2}=1$, the variance reduction for ${\bar{y}}_{\mathrm{II}}$ is maximized due to the strong correlation between auxiliary and study variables.

### 3.4 Regression estimator

Design and analysis strategies can be integrated: Fuller (2009b) combined rejective sampling and regression adjustment in surveys, and Li and Ding (2020) combined rerandomization and regression adjustment in experiments. Under TPRS, a regression estimator is


(10) $${\bar{y}}_{\mathrm{II},\mathrm{reg}}={\bar{y}}_{\mathrm{II}}-{\left({\bar{x}}_{\mathrm{II}}-{\bar{x}}_{I}\right)}^{T}{\hat{\beta }}_{\mathrm{II}},$$

with ${\hat{\beta }}_{\mathrm{II}}$ specified earlier in (1). To derive the asymptotic distribution of ${\bar{y}}_{\mathrm{II},\mathrm{reg}}$ in TPRS, we decompose it as follows:


(11) $$\begin{array}{rl} n_{\mathrm{II}}^{1/2}\left({\bar{y}}_{\mathrm{II},\mathrm{reg}}-{\bar{y}}_{0}\right) & =n_{\mathrm{II}}^{1/2}\left({\bar{y}}_{\mathrm{II}}-{\bar{y}}_{I}\right)-n_{\mathrm{II}}^{1/2}({\bar{x}}_{\mathrm{II}}-{\bar{x}}_{I}{)}^{T}\beta_{0}\\ & \;-n_{\mathrm{II}}^{1/2}({\bar{x}}_{\mathrm{II}}-{\bar{x}}_{I}{)}^{T}({\hat{\beta }}_{\mathrm{II}}-\beta_{0})+n_{\mathrm{II}}^{1/2}\left({\bar{y}}_{I}-{\bar{y}}_{0}\right)\\ & =n_{\mathrm{II}}^{1/2}\left({\bar{e}}_{\mathrm{II}}-{\bar{e}}_{I}\right)-n_{\mathrm{II}}^{1/2}({\bar{x}}_{\mathrm{II}}-{\bar{x}}_{I}{)}^{T}({\hat{\beta }}_{\mathrm{II}}-\beta_{0})+n_{\mathrm{II}}^{1/2}\left({\bar{y}}_{I}-{\bar{y}}_{0}\right). \end{array}$$

Here, $n_{\mathrm{II}}^{1/2}({\bar{e}}_{\mathrm{II}}-{\bar{e}}_{I})$ represents the error of *e* in the phase-II sample conditional on the phase-I sample, $n_{\mathrm{II}}^{1/2}({\bar{x}}_{\mathrm{II}}-{\bar{x}}_{I}{)}^{T}({\hat{\beta }}_{\mathrm{II}}-\beta_{0})$ is of order $o_{P}(1)$, and $n_{\mathrm{II}}^{1/2}({\bar{y}}_{I}-{\bar{y}}_{0})$ represents the error of *y* in the phase-I sample. The limiting distribution of ${\bar{y}}_{\mathrm{II},\mathrm{reg}}$ is given in the following theorem.

Theorem 2 Suppose Assumption 1 holds. Under TPRS in Definition 1, the regression estimator ${\bar{y}}_{\mathrm{II},\mathrm{reg}}$ in (10) has the following limiting distribution:

(12) $$n_{\mathrm{II}}^{1/2}({\bar{y}}_{\mathrm{II},\mathrm{reg}}-{\bar{y}}_{0})|F\to N\left\{0,f_{\mathrm{II},I}(1-f_{I,0})V_{yy}+(1-f_{\mathrm{II},I})V_{ee}\right\},$$

a.s., where $V_{uv}$ is defined in (5), $e=y-x^{T}\beta_{0}$, and $f_{\mathrm{II},I}$ and $f_{I,0}$ are defined in Assumption 1.

Fuller (2009b) provided the consistency and asymptotic variance of the regression estimator under single-phase rejective sampling, but did not include the asymptotic distribution results. The asymptotic distribution result in Theorem 2 complements Fuller (2009b)’s work, specifically by considering a census in phase I.

The limiting distribution of $n_{\mathrm{II}}^{1/2}({\bar{y}}_{\mathrm{II},\mathrm{reg}}-{\bar{y}}_{0})|F$ in (12) under TPRS does not depend on $\gamma^{2}$, and as $\gamma^{2}$ increases, it becomes equivalent to nonrejective sampling. This demonstrates the equivalence of the limiting distributions of ${\bar{y}}_{\mathrm{II},\mathrm{reg}}$ with and without rejective sampling, aligning with Fuller (2009b), who found similar performance of regression estimators in single-phase samples regardless of rejective sampling.

Theorems 1 and 2 highlight the role of rejective sampling in estimation. When $\gamma^{2}\approx 0$, the asymptotic design variance of the simple mean estimator ${\bar{y}}_{\mathrm{II}}$ is close to that of the two-phase regression estimator ${\bar{y}}_{\mathrm{II},\mathrm{reg}}$. That is, the simple mean estimator under rejective sampling performs similarly to the regression estimator.

If the two-phase regression estimator includes additional covariates (regressors) beyond the design covariates *x*, the results in Theorem 2 still apply by replacing *x* with the regressors, whether rejective sampling is used or not. Despite similar limiting properties with and without rejective sampling, Fuller (2009b) recommended rejective sampling for its practical benefits. We can express the regression estimator as a weighted average of the $y_{i}$’s in the phase-II sample ${\bar{y}}_{\mathrm{II},\mathrm{reg}}=\sum_{i\in B}\omega_{i}y_{i}$, where


$$\omega_{i}=1+{\left({\bar{x}}_{I}-{\bar{x}}_{\mathrm{II}}\right)}^{T}{\left\{\sum_{i\in B}{\left(x_{i}-{\bar{x}}_{\mathrm{II}}\right)}^{⊗2}\right\}}^{-1}\left(x_{i}-{\bar{x}}_{\mathrm{II}}\right)$$

are the weights. Without rejective sampling, $\omega_{i}$ may be negative due to influential values of $x_{i}$, which affects the robustness of the regression estimator. Rejective sampling reduces this chance, as demonstrated by simulation from Legg and Yu (2010) under single-phase sampling and further confirmed by our simulation under TPRS. However, rejective sampling does not completely eliminate the occurrence of negative weights. In these cases, one might consider alternative estimator, such as calibration weighting, as discussed in Section 1.

Moreover, rejective sampling improves the covariate balances in phase-II sample, evidenced by a smaller asymptotic design variance of $n_{\mathrm{II}}^{1/2}({\bar{x}}_{\mathrm{II}}-{\bar{x}}_{0})$ compared with designs without rejective sampling. Specially, under TPRS, the limit of the design covariance $a.\mathrm{cov}\{n_{\mathrm{II}}^{1/2}({\bar{x}}_{\mathrm{II}}-{\bar{x}}_{0})|Q_{I}<\gamma^{2}\}=\{(1-f_{\mathrm{II},I})v_{p,\gamma^{2}}+(f_{\mathrm{II},I}-f_{\mathrm{II},0})\}V_{xx}$ is always no larger than that without rejective sampling $a.\mathrm{cov}\{n_{\mathrm{II}}^{1/2}({\bar{x}}_{\mathrm{II}}-{\bar{x}}_{0})|F\}=(1-f_{\mathrm{II},0})V_{xx}$.

### 3.5 Inference: variance estimators and confidence intervals

To infer the population mean ${\bar{y}}_{0}$ based on Theorems 1 and 2, we first estimate the asymptotic design variances and covariances. Let the estimator for $V_{uv}$ be


$${\hat{V}}_{uv}=\frac{1}{n_{\mathrm{II}}-1}\sum_{i\in B}(u_{i}-{\bar{u}}_{\mathrm{II}})(v_{i}-{\bar{v}}_{\mathrm{II}}{)}^{T}.$$

Proposition 1 Under Assumption 1, ${\hat{V}}_{uv}$ is a consistent estimator of $V_{uv}$ under both two-phase simple random sampling and TPRS in Definition 1.

A consistent estimator for $R^{2}$ is ${\hat{R}}^{2}=({\hat{V}}_{yx}{\hat{V}}_{xx}^{-1}{\hat{V}}_{xy})/{\hat{V}}_{yy}$. The variance estimator for ${\bar{y}}_{\mathrm{II}}$ under TPRS is


$$\frac{1}{n_{\mathrm{II}}}\left[\left(1-\frac{n_{\mathrm{II}}}{n_{I}}\right)\left\{1-\left(1-v_{\;p,\gamma^{2}}\right){\hat{R}}^{2}\right\}+\frac{n_{\mathrm{II}}}{n_{I}}\left(1-\frac{n_{I}}{N}\right)\right]{\hat{V}}_{yy}.$$

Define ${\hat{e}}_{i}=y_{i}-x_{i}^{T}{\hat{\beta }}_{\mathrm{II}}$ for phase-II samples. Then, we can estimate $V_{ee}$ by


$${\hat{V}}_{ee}=\frac{1}{n_{\mathrm{II}}-p-1}\sum_{i\in B}({\hat{e}}_{i}-{\bar{e}}_{\mathrm{II}}{)}^{2},$$

where $n_{\mathrm{II}}-p-1$ adjusts for degrees of freedom due to estimating $\beta_{0}$. Decompose $V_{yy}$ into $\beta_{0}^{T}V_{xx}\beta_{0}+V_{ee}$ and estimate it by ${\hat{V}}_{yy}={\hat{\beta }}_{\mathrm{II}}^{T}{\hat{V}}_{xx}{\hat{\beta }}_{\mathrm{II}}+{\hat{V}}_{ee}$. A consistent variance estimator for ${\bar{y}}_{\mathrm{II},\mathrm{reg}}$ is


(13) $${\hat{V}}_{\mathrm{reg}}=\frac{1}{n_{\mathrm{II}}}\left\{\frac{n_{\mathrm{II}}}{n_{I}}\left(1-\frac{n_{I}}{N}\right){\hat{V}}_{yy}+\left(1-\frac{n_{\mathrm{II}}}{n_{I}}\right){\hat{V}}_{ee}\right\}.$$

We can construct the asymptotic (1−α) confidence interval of ${\bar{y}}_{0}$ based on ${\bar{y}}_{\mathrm{II}}$ as


$$\left({\bar{y}}_{\mathrm{II}}-n_{\mathrm{II}}^{-1/2}\nu_{1-\alpha /2}({\hat{R}}^{2}){\hat{V}}_{yy}^{1/2},{\bar{y}}_{\mathrm{II}}-n_{\mathrm{II}}^{-1/2}\nu_{\alpha /2}({\hat{R}}^{2}){\hat{V}}_{yy}^{1/2}\right),$$

where $\nu_{\alpha }(R^{2})$ as the 100αth quantile of the distribution of


$${\left(1-\frac{n_{\mathrm{II}}}{n_{I}}\right)}^{1/2}\left\{RL_{\;p,\gamma^{2}}+{\left(1-R^{2}\right)}^{1/2}Z_{2}\right\}+{\left\{\frac{n_{\mathrm{II}}}{n_{I}}\left(1-\frac{n_{I}}{N}\right)\right\}}^{1/2}Z_{3},$$

and the counterpart based on ${\bar{y}}_{\mathrm{II},\mathrm{reg}}$ as


$$\left({\bar{y}}_{\mathrm{II},\mathrm{reg}}-n_{\mathrm{II}}^{-1/2}{\hat{V}}_{\mathrm{reg}}^{1/2}z_{1-\alpha /2},{\bar{y}}_{\mathrm{II}}-n_{\mathrm{II}}^{-1/2}{\hat{V}}_{\mathrm{reg}}^{1/2}z_{\alpha /2}\right).$$

## 4 Two-phase rejective sampling with general sampling

### 4.1 Notation

Two-phase sampling is commonly used in national health surveys, such as the US National Health and Nutrition Examination Survey (Centers for Disease Control and Prevention, 2024) and the US National Health Interview Survey (National Center for Health Statistics, 2024). Complex sampling designs are often considered for both first- and second-phase samples, such as Poisson sampling designs, stratified multistage cluster sample designs and sampling with probability proportional to measures of size.

We now consider two-phase sampling with general phase-I and II designs. Let $\pi_{Ii}$ be the probability of including unit *i* in the phase-I sample A, and let $\pi_{\mathrm{II}i|A}$ be the conditional probability of including unit *i* in the phase-II sample B given that unit *i* is in the phase-I sample. Let $n_{I}$ and $n_{\mathrm{II}}$ be the sample sizes of the phase-I sample and the phase-II sample, respectively.

Define the finite population mean as ${\bar{u}}_{0}=N^{-1}\sum_{i=1}^{N}u_{i}$. With slight abuse of the notation, define the phase-I estimator and the phase-II estimator as


(14) $${\bar{u}}_{I}=\frac{1}{\sum_{i\in A}\pi_{Ii}^{-1}}\sum_{i\in A}\frac{u_{i}}{\pi_{Ii}}$$

and


(15) $${\bar{u}}_{\mathrm{II}}=\frac{1}{\sum_{i\in B}(\pi_{\mathrm{II}i}^{*}{)}^{-1}}\sum_{i\in B}\frac{u_{i}}{\pi_{\mathrm{II}i}^{*}},$$

respectively. The phase-I estimator is a Hájek estimator for ${\bar{u}}_{0}$, while the phase-II estimator, known as the double-expansion estimator (Kott & Stukel, 1997) or a $\pi^{*}$ estimator (Särndal et al., 2003), is generally not a Hájek estimator for ${\bar{u}}_{0}$ because $\pi_{\mathrm{II}i}^{*}=\pi_{Ii}\pi_{\mathrm{II}i|A}$ is not the probability of *i* being selected for phase II in general as we discussed in Section 2.1.

To calculate the design variances for (14) and (15), we require positive second-order inclusion probabilities. The probabilities, $\pi_{Iij}=P(i,j\in A|F)$ and $\pi_{\mathrm{II}ij|A}=P(i,j\in B|i,j\in A)$, determine the probability or the conditional probability of pairs of units being included in phase-I and phase-II samples, respectively.

Under suitable regularity conditions on sampling (with details in Section 4.2), the sums $\sum_{i\in A}\pi_{Ii}^{-1}$ and $\sum_{i\in B}(\pi_{\mathrm{II}i}^{*}{)}^{-1}$ are design consistent for the population size *N*. Using Taylor expansion and ignoring small order terms, the design covariance of ${\bar{u}}_{I}$ and ${\bar{v}}_{I}$ is


(16) $$V_{uv,0}=\mathrm{cov}\left({\bar{u}}_{I},{\bar{v}}_{I}|F\right)=\frac{1}{N^{2}}\sum_{i=1}^{N}\sum_{\;j=1}^{N}\frac{\pi_{Iij}-\pi_{Ii}\pi_{Ij}}{\pi_{Ii}\pi_{Ij}}(u_{i}-{\bar{u}}_{0})(v_{\;j}-{\bar{v}}_{0}{)}^{T},$$

and the conditional design covariance of ${\bar{u}}_{\mathrm{II}}$ and ${\bar{v}}_{\mathrm{II}}$ given the phase-I sample is


(17) $$V_{uv,I}=\mathrm{cov}\left({\bar{u}}_{\mathrm{II}},{\bar{v}}_{\mathrm{II}}|A,F\right)=\frac{1}{N^{2}}\sum_{i\in A}\sum_{\;j\in A}\frac{\pi_{\mathrm{II}ij|A}-\pi_{\mathrm{II}i|A}\pi_{\mathrm{II}j|A}}{\pi_{\mathrm{II}i}^{*}\pi_{\mathrm{II}j}^{*}}(u_{i}-{\bar{u}}_{I})(v_{\;j}-{\bar{v}}_{I}{)}^{T}.$$

We assume that $V_{xx,0}$ and $V_{xx,I}$ are positive definite for all phase-I samples.

### 4.2 Two-phase rejective sampling with general sampling

We define TPRS with general sampling designs as follows.

Definition 2 **TPRS with general sampling**

Two-phase rejective sampling with general sampling consists of two steps:

Step 1. Select a phase-I sample A by a general *π* sampling with the inclusion probability $\pi_{Ii}$. For i∈A, record $x_{i}$.

Step 2. Treat the phase-I sample A as the population and select a phase-II sample B by a general *π* sampling with the conditional inclusion probability $\pi_{\mathrm{II}i|A}$. Accept the phase-II sample if

$$Q_{I}=({\bar{x}}_{\mathrm{II}}-{\bar{x}}_{I}{)}^{T}V_{xx,I}^{-1}({\bar{x}}_{\mathrm{II}}-{\bar{x}}_{I})<\gamma^{2},$$

where $\gamma^{2}>0$ is a prespecified constant, and $V_{xx,I}$ is the design variance of ${\bar{x}}_{\mathrm{II}}-{\bar{x}}_{I}$ given A, given by (17) with *u* and *v* being *x*. For i∈B, record $y_{i}$.

For the population mean ${\bar{y}}_{0}$, the $\pi^{*}$ estimator is


(18) $${\bar{y}}_{\mathrm{II}}=\frac{1}{\sum_{i\in B}(\pi_{\mathrm{II}i}^{*}{)}^{-1}}\sum_{i\in B}\frac{y_{i}}{\pi_{\mathrm{II}i}^{*}},$$

recalling that $\pi_{\mathrm{II}i}^{*}=\pi_{Ii}\pi_{\mathrm{II}i|A}$. We focus on the $\pi^{*}$ estimator for simplicity because the REE reviewed in Section 2.1 is more natural for two-phase stratified sampling. Below, we will show that integrating the $\pi^{*}$ estimator with TPRS suffices to attain favourable design properties.

### 4.3 Large-sample design properties

To understand the limiting properties of ${\bar{y}}_{\mathrm{II}}$, we follow the asymptotic framework in Section 3 and specify the following regularity conditions for TPRS in Definition 2.

Assumption 2 Assumption 1(i) and (ii) hold. (iii) The phase-I estimator (14) satisfies

$$\mathrm{var}({\bar{u}}_{I}|F{)}^{-1/2}({\bar{u}}_{I}-{\bar{u}}_{0})|F\to N(0,1)\;a.s.,$$

with $\mathrm{var}(n_{I}^{1/2}{\bar{u}}_{I}|F)=O_{P}(1)$, where *u* represents components of either *x* or *y*.

(iv) The sequence of phase-I selection probabilities are bounded by $K_{I,\text{L}}<n_{I}^{-1}N\pi_{Ii}<K_{I,\text{U}}$ for all *i*, for some positive $K_{I,\text{L}}>0$ and $K_{I,U}>0$, and the design weighted sums of moments converge to constants,

$$\lim_{N\to \infty }\sum_{i\in A}\pi_{Ii}^{-1}(1,x_{i}^{T},y_{i},y_{i}^{2}{)}^{T}(1,x_{i}^{T},y_{i},y_{i}^{2})=M_{I}\;a.s.,$$

where $M_{I}$ is a matrix of constants.

(v) The phase-II estimator (15) satisfies

$$\mathrm{var}({\bar{u}}_{\mathrm{II}}|A,F{)}^{-1/2}({\bar{u}}_{\mathrm{II}}-{\bar{u}}_{I})|A,F\to N(0,1)\;a.s.,$$

and $\mathrm{var}(n_{\mathrm{II}}^{1/2}{\bar{u}}_{\mathrm{II}}|A,F)=O_{P}(1)$;

(vi) The sequence of phase-II selection probabilities are bounded by $K_{\mathrm{II},\text{L}}<n_{\mathrm{II}}^{-1}n_{I}\pi_{\mathrm{II}i|A}<K_{\mathrm{II},\text{U}}$ for all *i*, for some positive $K_{\mathrm{II},\text{L}}>0$ and $K_{\mathrm{II},\text{U}}>0$, and the design weighted sums of moments converge to constants,

$$\lim_{N\to \infty }\sum_{i\in B}\pi_{\mathrm{II}i|A}^{-1}(1,x_{i}^{T},y_{i},y_{i}^{2}{)}^{T}(1,x_{i}^{T},y_{i},y_{i}^{2})=M_{\mathrm{II}}\;a.s.,$$

where $M_{\mathrm{II}}$ is a matrix of constants.

(vii) The design covariance between the differences in *x* and *e* in phases I and II is negligible: $\mathrm{cov}({\bar{x}}_{\mathrm{II}}-{\bar{x}}_{I},{\bar{e}}_{\mathrm{II}}-{\bar{e}}_{I}|A,F)=o_{P}(n_{\mathrm{II}}^{-1})$.

The conditions in Assumption 2 are standard for sample moments and sampling designs (Fuller, 2009a, Theorem 3.3.1). They ensure the general applicability of the phase-I and phase-II estimators across various designs (Chapter 3, Fuller, 2009a). For instance, Assumption 2(vii) holds under two-phase simple random sampling and stratified sampling. A heuristic explanation is provided below. Let $r_{i}=y_{i}-x_{i}^{T}{\hat{\beta }}_{I}$ be the residual based on the phase-I regression had the study variables been measured in the phase-I sample, where ${\hat{\beta }}_{I}=\{\sum_{i\in A}\pi_{Ii}^{-1}(x_{i}-{\bar{x}}_{I}{)}^{⊗2}{\}}^{-1}\sum_{i\in A}\pi_{Ii}^{-1}(x_{i}-{\bar{x}}_{I})(y_{i}-{\bar{y}}_{I})$ is the phase-I regression coefficient. Then, we have $\mathrm{cov}({\bar{x}}_{\mathrm{II}}-{\bar{x}}_{I},{\bar{r}}_{\mathrm{II}}-{\bar{r}}_{I}|A,F)=0$. Recall that $e_{i}=y_{i}-x_{i}^{T}\beta_{0}$, which can be written as $e_{i}=r_{i}+x_{i}^{T}({\hat{\beta }}_{I}-\beta_{0})$. Thus, we have


(19) $$\begin{array}{rl} \mathrm{cov}({\bar{x}}_{\mathrm{II}}-{\bar{x}}_{I},{\bar{e}}_{\mathrm{II}}-{\bar{e}}_{I}|A,F) & =\mathrm{cov}({\bar{x}}_{\mathrm{II}}-{\bar{x}}_{I},{\bar{r}}_{\mathrm{II}}-{\bar{r}}_{I}|A,F)\\ & \;+\mathrm{cov}\{{\bar{x}}_{\mathrm{II}}-{\bar{x}}_{I},({\bar{x}}_{\mathrm{II}}-{\bar{x}}_{I}{)}^{T}({\hat{\beta }}_{I}-\beta_{0})|A,F\}\\ & =\mathrm{var}({\bar{x}}_{\mathrm{II}}-{\bar{x}}_{I}|A,F)({\hat{\beta }}_{I}-\beta_{0}). \end{array}$$

Because $\mathrm{var}({\bar{x}}_{\mathrm{II}}-{\bar{x}}_{I}|A,F)=O_{P}(n_{\mathrm{II}}^{-1})$  a.s., where the probability distribution in $O_{P}$ is induced by phase-II sampling given (A,F), and ${\hat{\beta }}_{I}-\beta_{0}=O_{P}(n_{I}^{-1})$, where the probability distribution in $O_{P}$ is induced by phase-I sampling, the quantity in (19) is of order $o_{P}(n_{\mathrm{II}}^{-1})$.

Following the decomposition in (6), we decompose $n_{\mathrm{II}}^{1/2}({\bar{y}}_{\mathrm{II}}-{\bar{y}}_{0})$ into three components


(20) $$n_{\mathrm{II}}^{1/2}\left({\bar{y}}_{\mathrm{II}}-{\bar{y}}_{0}\right)=T_{1}+T_{2}+T_{3},$$

where $T_{1}=n_{\mathrm{II}}^{1/2}({\bar{x}}_{\mathrm{II}}-{\bar{x}}_{I}{)}^{T}\beta_{0}$, $T_{2}=n_{\mathrm{II}}^{1/2}({\bar{e}}_{\mathrm{II}}-{\bar{e}}_{I})$, and $T_{3}=n_{\mathrm{II}}^{1/2}({\bar{y}}_{I}-{\bar{y}}_{0})$ with the general *π* estimators for ${\bar{u}}_{I}$ and ${\bar{u}}_{\mathrm{II}}$ defined in (14) and (15), respectively. The limiting distribution of $\left(T_{1},T_{2},T_{3}\right)$ is given in the following lemma.

Lemma 2 Suppose Assumption 2 holds. Without the rejection step in TPRS in Definition 2, the joint distribution of $\left(T_{1},T_{2},T_{3}\right)$ in the decomposition (20) has the following limiting distribution:

$$\left(\begin{array}{c} T_{1}\\ T_{2}\\ T_{3} \end{array}\right)|F\to N\left\{\left(\begin{array}{c} 0\\ 0\\ 0 \end{array}\right),\left(\begin{array}{ccc} V_{1}, & 0 & 0\\ 0 & V_{2} & 0\\ 0 & 0 & V_{3} \end{array}\right)\right\},$$

a.s. for all sequences of finite populations, where

(21) $$V_{1}=\lim_{N\to \infty }n_{\mathrm{II}}\beta_{0}^{T}E\left(V_{xx,I}|F\right)\beta_{0},$$

(22) $$V_{2}=\lim_{N\to \infty }n_{\mathrm{II}}E\left(V_{ee,I}|F\right),$$

(23) $$V_{3}=\lim_{N\to \infty }n_{\mathrm{II}}V_{yy,0}.$$

By construction, the distribution of $n_{\mathrm{II}}^{1/2}({\bar{y}}_{\mathrm{II}}-{\bar{y}}_{0})$ under TPRS in Definition 2 is equivalent to the distribution of $n_{\mathrm{II}}^{1/2}({\bar{y}}_{\mathrm{II}}-{\bar{y}}_{0})|(Q_{I}<\gamma^{2})$ without rejective sampling. To study the asymptotic design property, we define $D_{I}=(n_{\mathrm{II}}V_{xx,I}{)}^{-1/2}n_{\mathrm{II}}^{1/2}({\bar{x}}_{\mathrm{II}}-{\bar{x}}_{I})$. Then, $Q_{I}$ is expressed as $D_{I}^{T}D_{I}$, with $D_{I}\to N(0,I_{\;p})$ and thus $D_{I}^{T}D_{I}\to \chi_{\;p}^{2}$ a.s.

Theorem 3 Suppose Assumption 2 holds. Under TPRS in Definition 2, ${\bar{y}}_{\mathrm{II}}$ follows the limiting distribution:

(24) $$n_{\mathrm{II}}^{1/2}({\bar{y}}_{\mathrm{II}}-{\bar{y}}_{0})|\left(Q_{I}<\gamma^{2}\right)\to V_{1}^{1/2}L_{\;p,\gamma^{2}}+V_{2}^{1/2}Z_{1}+V_{3}^{1/2}Z_{2},$$

where $V_{1}$, $V_{2},$ and $V_{3}$ are defined in (21)–(23), $Z_{1}$ and $Z_{2}$ are standard normal variables, and $\left(L_{\;p,\gamma^{2}},Z_{1},Z_{2}\right)$ are jointly independent.

The results of TPRS with general sampling are similar to those of TPRS with simple random sampling, hence inheriting all the benefits, including enhanced covariate balance, and reduced variance and quantile range, as detailed in Section 3.

### 4.4 Regression estimator

Integrating the design and analysis strategies, the two-phase regression estimator for ${\bar{y}}_{0}$ is


(25) $${\bar{y}}_{\mathrm{II},\mathrm{reg}}={\bar{y}}_{\mathrm{II}}-{\left({\bar{x}}_{\mathrm{II}}-{\bar{x}}_{I}\right)}^{T}{\hat{\beta }}_{\mathrm{II}},$$

where


$${\hat{\beta }}_{\mathrm{II}}={\left\{\sum_{i\in B}\frac{{\left(x_{i}-{\bar{x}}_{\mathrm{II}}\right)}^{⊗2}}{\pi_{\mathrm{II}i}^{*}}\right\}}^{-1}\sum_{i\in B}\frac{\left(x_{i}-{\bar{x}}_{\mathrm{II}})(y_{i}-{\bar{y}}_{\mathrm{II}}\right)}{\pi_{\mathrm{II}i}^{*}}$$

is the regression coefficient based on the phase-II sample.

Theorem 4 Suppose Assumption 2 holds. Under TPRS in Definition 2, the regression estimator ${\bar{y}}_{\mathrm{II},\mathrm{reg}}$ in (25) has the following limiting distribution:

$$n_{\mathrm{II}}^{1/2}({\bar{y}}_{\mathrm{II},\mathrm{reg}}-{\bar{y}}_{0})|F\to N\left(0,V_{\mathrm{II},\mathrm{reg}}\right),$$

a.s. for all sequences of finite populations, where

$$V_{\mathrm{II},\mathrm{reg}}=\lim_{N\to \infty }n_{\mathrm{II}}\left\{V_{yy,0}+E\left(V_{ee,I}|F\right)\right\}$$

with $V_{yy,0}$ and $V_{ee,I}$ defined in (16) and (17), respectively.

Theorem 4 indicates that the advantages of TPRS are also applicable to the regression estimator in a general setup.

### 4.5 Inference: variance estimators and confidence intervals

We estimate the asymptotic design variances of ${\bar{y}}_{\mathrm{II}}$ and ${\bar{y}}_{\mathrm{II},\mathrm{reg}}$ in general TPRS. For ${\bar{y}}_{\mathrm{II}}$, the variance is estimated by $n_{\mathrm{II}}^{-1}({\hat{V}}_{1}v_{p,\gamma^{2}}+{\hat{V}}_{2}+{\hat{V}}_{3}),$ where


(26) $$\begin{array}{rl} {\hat{V}}_{1} & =n_{\mathrm{II}}{\hat{\beta }}_{\mathrm{II}}^{T}V_{xx,I}{\hat{\beta }}_{\mathrm{II}},\\ {\hat{V}}_{2} & =\frac{n_{\mathrm{II}}}{N^{2}}\sum_{i\in B}\sum_{\;j\in B}\frac{\pi_{\mathrm{II}ij|A}-\pi_{\mathrm{II}i|A}\pi_{\mathrm{II}j|A}}{\pi_{\mathrm{II}i}^{*}\pi_{\mathrm{II}j}^{*}}\frac{({\hat{e}}_{i}-{\hat{e}}_{\mathrm{II}})({\hat{e}}_{\;j}-{\hat{e}}_{\mathrm{II}}{)}^{T}}{\pi_{\mathrm{II}ij|A}}, \end{array}$$

(27) $${\hat{V}}_{3}=\frac{n_{\mathrm{II}}}{N^{2}}\sum_{i\in B}\sum_{\;j\in B}\frac{\pi_{Iij}-\pi_{Ii}\pi_{Ij}}{\pi_{Ii}\pi_{Ij}}\frac{(y_{i}-{\bar{y}}_{\mathrm{II}})(y_{\;j}-{\bar{y}}_{\mathrm{II}}{)}^{T}}{\pi_{Iij}\pi_{\mathrm{II}ij|A}},$$

and ${\hat{e}}_{i}=y_{i}-x_{i}^{T}{\hat{\beta }}_{\mathrm{II}}$ and ${\hat{e}}_{\mathrm{II}}$ are (18) with $y_{i}$ being ${\hat{e}}_{i}$. For ${\bar{y}}_{\mathrm{II},\mathrm{reg}}$, the variance is estimated by $n_{\mathrm{II}}^{-1}({\hat{V}}_{2}+{\hat{V}}_{3})$.

Remark 1 Regarding variance estimation in two-phase sampling, two points are noteworthy. First, obtaining the joint inclusion probabilities $\pi_{Iij}$ and $\pi_{\mathrm{II}ij|A}$ may be difficult in practice, and approximations may be needed. For example, Haziza and Beaumont (2005) and Beaumont et al. (2015) considered a simplified variance estimator which does not require the information on $\pi_{\mathrm{II}ij|A}$ and specified conditions under which the bias of the variance estimator is negligible.

Second, while Horvitz–Thompson-type variance estimators (26) and (27) are standard, they can be unstable and negative in unequal probability sampling. An alternative is the Sen–Yates–Grundy-type (Sen, 1953; Yates & Grundy, 1953) variance estimators

(28) $${\hat{V}}_{2,\mathrm{SYG}}=-\frac{1}{2}\frac{n_{\mathrm{II}}}{N^{2}}\sum_{i\in B}\sum_{\;j\in B}\frac{\pi_{\mathrm{II}ij|A}-\pi_{\mathrm{II}i|A}\pi_{\mathrm{II}j|A}}{\pi_{\mathrm{II}ij|A}}{\left(\frac{{\hat{e}}_{i}-{\hat{e}}_{\mathrm{II}}}{\pi_{\mathrm{II}i}^{*}}-\frac{{\hat{e}}_{\;j}-{\hat{e}}_{\mathrm{II}}}{\pi_{\mathrm{II}j}^{*}}\right)}^{⊗2},$$

(29) $${\hat{V}}_{3,\mathrm{SYG}}=-\frac{1}{2}\frac{n_{\mathrm{II}}}{N^{2}}\sum_{i\in B}\sum_{\;j\in B}\left(\pi_{Iij}-\pi_{Ii}\pi_{Ij}\right){\left(\frac{y_{i}-{\bar{y}}_{\mathrm{II}}}{\pi_{Ii}}-\frac{y_{\;j}-{\bar{y}}_{\mathrm{II}}}{\pi_{Ij}}\right)}^{⊗2}.$$

These estimators are asymptotically equivalent to ${\hat{V}}_{2}$ and ${\hat{V}}_{3}$ with additional mean zero terms but are more stable and nonnegative for various sampling designs with fixed sample sizes (Hidiroglou et al., 2009).

We can construct the asymptotic (1−α) confidence interval for ${\bar{y}}_{0}$ based on ${\bar{y}}_{\mathrm{II}}$ as


$$\left({\bar{y}}_{\mathrm{II}}-n_{\mathrm{II}}^{-1/2}\nu_{1-\alpha /2}({\hat{V}}_{1},{\hat{V}}_{2},{\hat{V}}_{3}),\;{\bar{y}}_{\mathrm{II}}-n_{\mathrm{II}}^{-1/2}\nu_{\alpha /2}({\hat{V}}_{1},{\hat{V}}_{2},{\hat{V}}_{3})\right),$$

where $\nu_{\alpha }(V_{1},V_{2},V_{3})$ as the 100αth quantile of the distribution of $V_{1}^{1/2}L_{\;p,\gamma^{2}}+V_{2}^{1/2}Z_{1}+V_{3}^{1/2}Z_{2}$, and the counterpart based on ${\bar{y}}_{\mathrm{II},\mathrm{reg}}$ as


$$\left({\bar{y}}_{\mathrm{II},\mathrm{reg}}-n_{\mathrm{II}}^{-1/2}{\left({\hat{V}}_{2}+{\hat{V}}_{3}\right)}^{1/2}z_{1-\alpha /2},\;{\bar{y}}_{\mathrm{II},\mathrm{reg}}-n_{\mathrm{II}}^{-1/2}{\left({\hat{V}}_{2}+{\hat{V}}_{3}\right)}^{1/2}z_{\alpha /2}\right).$$

## 5 Extensions

### 5.1 Other designs

When covariates have varying importance, applying different thresholds for different covariates in rejective sampling can be more effective. Legg and Yu (2010) explored two approaches for this: weighted rejective sampling and sequential rejective sampling, with the former also studied by Lu et al. (2023) and the latter by Morgan and Rubin (2015) and Li et al. (2018) in rerandomization in experiments. We further develop sequential rejective sampling in two-phase sampling in Section S1 of the online supplementary material and demonstrate that it provides better balance control for each specific covariate compared with weighted rejective sampling.

Moreover, we introduce multi-phase rejective sampling and establish asymptotic design properties of the double-expansion estimator and the regression estimator in Section S2 of the online supplementary material. As a special case, if phase I is a census, the three-phase rejective sampling reduces to TPRS with rejective sampling in both phases. In both sequential and multi-phase rejective sampling methods, we employ block-wise Gram–Schmidt orthogonalization on tiers or phases of covariates. This strategy ensures a clear distinction between various sets of covariates, categorizing them according to their level of importance or their availability across different phases.

### 5.2 General parameters

The current framework can be extended to deal with general parameters defined by estimating equations. Let the general population parameter $\xi_{0}$ be defined as the solution to


$${\bar{s}}_{0}(\xi )=N^{-1}\sum_{i=1}^{N}s(y_{i};\xi )=0,$$

where $s(y_{i};\xi )$ is the *q*-dimensional estimating function of *ξ*. For simplicity, we also denote $s(y_{i};\xi )$ by $s_{i}(\xi )$. These parameters are general and encompass many parameters of interest in survey sampling. For example, if $s_{i}(\xi )=y_{i}-\xi$, $\xi_{0}$ is the population mean of *y*; if $s_{i}(\xi )=I(y_{i}<c)-\xi$ for some constant *c*, $\xi_{0}$ is the population proportion of *y* less than *c*; and if $s_{i}(\xi )=\{y_{i}-\xi_{1},(y_{i}-\xi_{1}{)}^{2}-\xi_{2}{\}}^{T}$, where $\xi =(\xi_{1},\xi_{2}{)}^{T}$, $\xi_{2,0}$ is the population variance of *y*. These estimating functions are differentiable with respect to *ξ*. However, nondifferentiable estimating equations can also be considered. For example, in quantile estimation, let $s_{i}(\xi )=I(y_{i}\leq \xi )-\tau$ for τ∈(0,1), then $\xi_{0}=\inf\{\xi \;:\;{\bar{s}}_{0}(\xi )\geq 0\}$ is the population 100τth quantile.

We focus on the phase-I and phase-II estimators (14) and (15) of ${\bar{s}}_{0}(\xi )$ in general TPRS, denoted as ${\bar{s}}_{I}(\xi )$ and ${\bar{s}}_{\mathrm{II}}(\xi )$, respectively. Let ${\bar{\xi }}_{\mathrm{II}}$ be the solution to ${\bar{s}}_{\mathrm{II}}(\xi )=0$.

Theorem 5 Suppose Assumption 2 holds for $s_{i}(\xi_{0})$ and that the regularity conditions in Assumption S2 hold. Under TPRS in Definition 2, ${\bar{\xi }}_{\mathrm{II}}$ follows the limiting distribution:

$$n_{\mathrm{II}}^{1/2}({\bar{\xi }}_{\mathrm{II}}-\xi_{0})|\left(Q_{I}<\gamma^{2}\right)\to \Gamma_{s}^{T}(V_{1}^{s}{)}^{1/2}L_{\;p,\gamma^{2}}+\Gamma_{s}^{T}(V_{2}^{s}{)}^{1/2}Z_{1}+\Gamma_{s}^{T}(V_{3}^{s}{)}^{1/2}Z_{2},$$

as $n_{\mathrm{II}}\to \infty$, where $\Gamma_{s}=\partial s_{0}(\xi_{0})/\partial \xi$ with $s_{0}(\xi )$ being the limiting function of ${\bar{s}}_{0}(\xi )$,

(30) $$V_{1}^{s}=\lim_{N\to \infty }n_{\mathrm{II}}B_{0}^{T}E\left(V_{xx,I}|F\right)B_{0},$$

(31) $$V_{2}^{s}=\lim_{N\to \infty }n_{\mathrm{II}}E\left(V_{e^{s}e^{s},I}|F\right),$$

(32) $$V_{3}^{s}=\lim_{N\to \infty }n_{\mathrm{II}}V_{ss,0},$$

$e_{i}^{s}=s_{i}-(x_{i}^{T}B_{0}{)}^{T},$  $B_{0}=\{\sum_{i=1}^{N}(x_{i}-{\bar{x}}_{0}{)}^{⊗2}{\}}^{-1}\sum_{i=1}^{N}(x_{i}-{\bar{x}}_{0})\{s_{i}(\xi_{0})-{\bar{s}}_{0}(\xi_{0}){\}}^{T}$, $Z_{1}$ and $Z_{2}$ are standard normal variables, and $\left(L_{\;p,\gamma^{2}},Z_{1},Z_{2}\right)$ are jointly independent.

Theorem 5 includes Theorem 3 as a special case. If $s_{i}(\xi )=y_{i}-\xi$, then $\Gamma_{s}=1$, and $V_{1}^{s},V_{2}^{s}$ and $V_{3}^{s}$ in (30)–(32) equal $V_{1},V_{2}$ and $V_{3}$ in (21)–(23).

We estimate the asymptotic design variances of ${\bar{\xi }}_{\mathrm{II}}$ similarly in Section 4.5. Specifically, the variance of ${\bar{\xi }}_{\mathrm{II}}$ can be estimated by $n_{\mathrm{II}}^{-1}{\hat{\Gamma }}_{s}^{T}({\hat{V}}_{1}^{s}v_{\;p,\gamma^{2}}+{\hat{V}}_{2}^{s}+{\hat{V}}_{3}){\hat{\Gamma }}_{s},$ where ${\hat{\Gamma }}_{s}$ is (15) with $u_{i}=\partial s(y_{i};\xi )/\partial \xi {|}_{\xi ={\bar{\xi }}_{\mathrm{II}}}$. Here, we have


$$\begin{array}{rl} {\hat{V}}_{1}^{s} & =n_{\mathrm{II}}{\hat{B}}_{\mathrm{II}}^{T}V_{xx,I}{\hat{B}}_{\mathrm{II}},\\ {\hat{V}}_{2}^{s} & =\frac{n_{\mathrm{II}}}{N^{2}}\sum_{i\in B}\sum_{\;j\in B}\frac{\pi_{\mathrm{II}ij|A}-\pi_{\mathrm{II}i|A}\pi_{\mathrm{II}j|A}}{\pi_{\mathrm{II}i}^{*}\pi_{\mathrm{II}j}^{*}}\frac{({\hat{e}}_{i}^{s}-{\hat{e}}_{\mathrm{II}}^{s})({\hat{e}}_{\;j}^{s}-{\hat{e}}_{\mathrm{II}}^{s}{)}^{T}}{\pi_{\mathrm{II}ij|A}},\\ {\hat{V}}_{3}^{s} & =\frac{n_{\mathrm{II}}}{N^{2}}\sum_{i\in B}\sum_{\;j\in B}\frac{\pi_{Iij}-\pi_{Ii}\pi_{Ij}}{\pi_{Ii}\pi_{Ij}}\frac{\{s_{i}-{\bar{s}}_{\mathrm{II}}({\bar{\xi }}_{\mathrm{II}})\}\{s_{\;j}-{\bar{s}}_{\mathrm{II}}({\bar{\xi }}_{\mathrm{II}}){\}}^{T}}{\pi_{Iij}\pi_{\mathrm{II}ij|A}}, \end{array}$$

where $s_{i}=s_{i}({\bar{\xi }}_{\mathrm{II}})$ for simplicity, ${\hat{B}}_{\mathrm{II}}=\{\sum_{i=1}^{N}(x_{i}-{\bar{x}}_{0}{)}^{⊗2}{\}}^{-1}\sum_{i=1}^{N}(x_{i}-{\bar{x}}_{0})\{s_{i}-{\bar{s}}_{\mathrm{II}}({\bar{\xi }}_{\mathrm{II}}){\}}^{T}$, ${\hat{e}}_{i}^{s}=s_{i}-(x_{i}^{T}{\hat{B}}_{\mathrm{II}}{)}^{T}$, and ${\hat{e}}_{\mathrm{II}}^{s}$ are (18) with $y_{i}$ being ${\hat{e}}_{i}^{s}$. Confidence intervals for components of $\xi_{0}$ can be constructed in a similar manner as in Section 4.5.

## 6 Empirical studies

We evaluate the finite-sample performance of rejective sampling through simulation. We first apply the TPRS with simple random sampling in Section 6.1. We then apply the three-phase rejective sampling based on an actual study in Section 6.2 to showcase the effectiveness of multiple phases.

### 6.1 Two-phase rejective sampling with simple random sampling

Consider the finite population with bivariate data $\left\{(x_{i},y_{i})\;:\;i=1,\ldots ,N=10^{5}\right\}$, where $x_{i}∼0.5^{1/2}(\chi_{1}^{2}-1)$ and $y_{i}=1+\beta x_{i}+e_{i}$ with $e_{i}∼N(0,1)$ and β∈{0.5,1,2}. This setup yields $R^{2}=\beta^{2}/(\beta^{2}+1)$ values of {0.2,0.5,0.8}.

We implement two-phase simple random sampling with phase-I and phase-II sample sizes of $n_{I}=5,000$ and $n_{\mathrm{II}}=200$, respectively, and the corresponding TPRS with various $\gamma^{2}\in \{0.01,0.05,0.1\}$. We compare the mean estimators ${\bar{y}}_{\mathrm{II}}$ and ${\bar{y}}_{\mathrm{II},\mathrm{reg}}$ in both sampling scenarios.

We summarize the results in Tables 1 and 2. In all scenarios, ${\bar{y}}_{\mathrm{II}}$ with rejective sampling improves the efficiency of ${\bar{y}}_{\mathrm{II}}$ without rejective sampling with the percentage of variance reduction increasing with $R^{2}$ and decreasing with $\gamma^{2}$, which is cohesive with our theoretical results in Theorem 1 and Corollary 1. Table 2 presents the theoretical values of percentage of variance reduction (9) under the simulation setup. The results in Table 1 are consistent with that in Table 2. The performance of ${\bar{y}}_{\mathrm{II}}$ with rejective sampling is close to that of ${\bar{y}}_{\mathrm{II},\mathrm{reg}}$, and the results for ${\bar{y}}_{\mathrm{II},\mathrm{reg}}$ in both rejective and nonrejective samples are similar, aligning with our theoretical results in Theorem 2. The variance reduction percentages for ${\bar{y}}_{\mathrm{II},\mathrm{reg}}$ under rejective sampling can be slightly negative, potentially due to finite sample sizes. Moreover, the regression weights in ${\bar{y}}_{\mathrm{II},\mathrm{reg}}$ can be negative for 30 simulated datasets under two-phase nonrejective sampling; however, the regression weights are always positive with rejective sampling in this simulation setting. This demonstrates the practical value of rejective sampling. Additionally, our variance estimators and confidence intervals, based on the asymptotic variance formula, prove accurate in both variance estimation and coverage rates, as evidenced in Table 2.

**Table 1.** Simulation results based on 1,000 Monte Carlo samples: bias (Bias $\times 10^{-2}$), variance (Var $\times 10^{-3}$), mean squared error (MSE $\times 10^{-3}$), variance estimate (VE $\times 10^{-3}$), and coverage rate (Cvg %) for 95% confidence intervals calculated based on the asymptotic variance formula, and the percentage of variance reduction of the estimator (VarRed %) under rejective sampling compared to the corresponding estimator under two-phase sample random sampling

	Method	Bias	Var	MSE	VE	Cvg	VarRed
$\gamma^{2}$		$\left(\times 10^{-2}\right)$	$\left(\times 10^{-3}\right)$	$\left(\times 10^{-3}\right)$	$\left(\times 10^{-3}\right)$	(%)	(%)
β=0.5 and $R^{2}=0.2$							
∞	${\bar{y}}_{\mathrm{II}}$	− 0.30	6.0	12.1	6.2	95.5	–
	${\bar{y}}_{\mathrm{II},\mathrm{reg}}$	− 0.22	4.9	9.9	5.0	95.3	–
0.01	${\bar{y}}_{\mathrm{II}}$	0.35	5.0	10.0	5.0	95.4	17
	${\bar{y}}_{\mathrm{II},\mathrm{reg}}$	0.35	5.0	10.1	5.0	95.3	− 2
0.05	${\bar{y}}_{\mathrm{II}}$	− 0.26	5.1	10.2	5.0	94.5	16
	${\bar{y}}_{\mathrm{II},\mathrm{reg}}$	− 0.27	5.1	10.2	5.0	94.3	− 3
0.1	${\bar{y}}_{\mathrm{II}}$	− 0.07	5.1	10.1	5.1	95.0	16
	${\bar{y}}_{\mathrm{II},\mathrm{reg}}$	− 0.05	5.0	10.1	5.0	95.2	− 2
β=1 and $R^{2}=0.5$							
∞	${\bar{y}}_{\mathrm{II}}$	0.00	10.1	20.3	10.0	94.3	–
	${\bar{y}}_{\mathrm{II},\mathrm{reg}}$	0.12	5.5	11.0	5.2	94.1	–
0.01	${\bar{y}}_{\mathrm{II}}$	− 0.05	5.1	10.1	5.1	94.8	50
	${\bar{y}}_{\mathrm{II},\mathrm{reg}}$	− 0.05	5.0	10.1	5.2	95.1	8
0.05	${\bar{y}}_{\mathrm{II}}$	− 0.09	5.3	10.6	5.2	94.5	48
	${\bar{y}}_{\mathrm{II},\mathrm{reg}}$	− 0.09	5.2	10.4	5.2	94.6	5
0.1	${\bar{y}}_{\mathrm{II}}$	− 0.17	5.3	10.7	5.3	94.5	47
	${\bar{y}}_{\mathrm{II},\mathrm{reg}}$	− 0.19	5.2	10.5	5.2	94.7	4
β=2 and $R^{2}=0.8$							
∞	${\bar{y}}_{\mathrm{II}}$	− 0.26	24.7	49.4	25.1	94.7	–
	${\bar{y}}_{\mathrm{II},\mathrm{reg}}$	0.00	6.0	12.0	5.8	94.3	–
0.01	${\bar{y}}_{\mathrm{II}}$	0.08	5.8	11.7	5.7	94.8	76
	${\bar{y}}_{\mathrm{II},\mathrm{reg}}$	0.06	5.8	11.5	5.8	94.8	4
0.05	${\bar{y}}_{\mathrm{II}}$	− 0.35	6.0	12.1	5.9	94.9	76
	${\bar{y}}_{\mathrm{II},\mathrm{reg}}$	− 0.30	5.6	11.2	5.8	95.2	7
0.1	${\bar{y}}_{\mathrm{II}}$	0.10	6.4	12.8	6.2	94.8	74
	${\bar{y}}_{\mathrm{II},\mathrm{reg}}$	0.09	5.9	11.7	5.8	95.0	3

*Note.*  $R^{2}=\beta^{2}/(\beta^{2}+1)\in \{0.2,0.5,0.8\}.$

**Table 2.** Theoretical values of percentage of variance reduction under the simulation setup

	$\gamma^{2}$	0.01	0.05	0.1
	$v_{\;p,\gamma^{2}}$	0.003	0.017	0.033
$\frac{1-f_{\mathrm{II},I}}{1-f_{\mathrm{II},I}f_{I,F}}(1-v_{\;p,\gamma^{2}})R^{2}$	$R^{2}=0.2$	19.2	47.9	76.7
	$R^{2}=0.5$	18.9	47.3	75.7
	$R^{2}=0.8$	18.6	46.5	74.4

### 6.2 Three-phase sampling based on the academic performance index data

To demonstrate the practical relevance, we use the Academic Performance Index (API) data. The full population data consist of 6,194 observations for all California schools with at least 100 students based on standardized testing of students. We use the API in year 2000 as the study variable *y*, the API in year 1999 as the auxiliary variable *x*, and the percentage of English Language Learners, the percentage of students eligible for subsidized meals, the percentage of students for whom this is the first year at the school as additional auxiliary variable *z* in both the design and analysis stages. The parameter of interest is the population mean of the API in year 2000.

We employed three-phase sampling designs. We select a phase-I sample A of size $n_{I}=2,000$ by simple random sampling. For i∈A, we observe $x_{i}$. We select a phase-II sample B of size $n_{\mathrm{II}}$ by Poisson sampling with $\pi_{\mathrm{II}i}=p_{\mathrm{II}i}E(n_{\mathrm{II}})$, where $E(n_{\mathrm{II}})$ is the expected sample size of the phase-II sample, $p_{\mathrm{II}i}\propto x_{i}$ and $\sum_{i\in A}p_{\mathrm{II}i}=1$. For i∈B, we observe $z_{i}$ and calculate $a_{i}=z_{i}-{\bar{z}}_{\mathrm{II}}-(x_{i}-{\bar{x}}_{\mathrm{II}}{)}^{T}{\hat{\beta }}_{zx,\mathrm{II}}$. We select a phase-III sample C by Poisson sampling with $\pi_{\mathrm{III}i}=p_{\mathrm{III}i}E(n_{\mathrm{III}}),$ where $E(n_{\mathrm{III}})$ is the expected sample size of the phase-III sample, $p_{\mathrm{III}i}$ is proportional to the summation of components in $z_{i}$ and $\sum_{i\in B}p_{\mathrm{III}i}=1$. For i∈C, we observe $y_{i}$. We consider the above three-phase sampling without rejective sampling and with rejective sampling described in Section S2.2 online supplementary material. We consider the impact of the number of phases, phase sample sizes $E(n_{\mathrm{II}})$ and $E(n_{\mathrm{III}})$, and constraints $\gamma_{1}^{2}$ and $\gamma_{2}^{2}$. For comparison, we also consider two-phase sampling designs. In a two-phase sampling, we assume that the outcome is measured for the phase-II samples. This may be more costly than the three-phase sampling.

**Figure 1.**
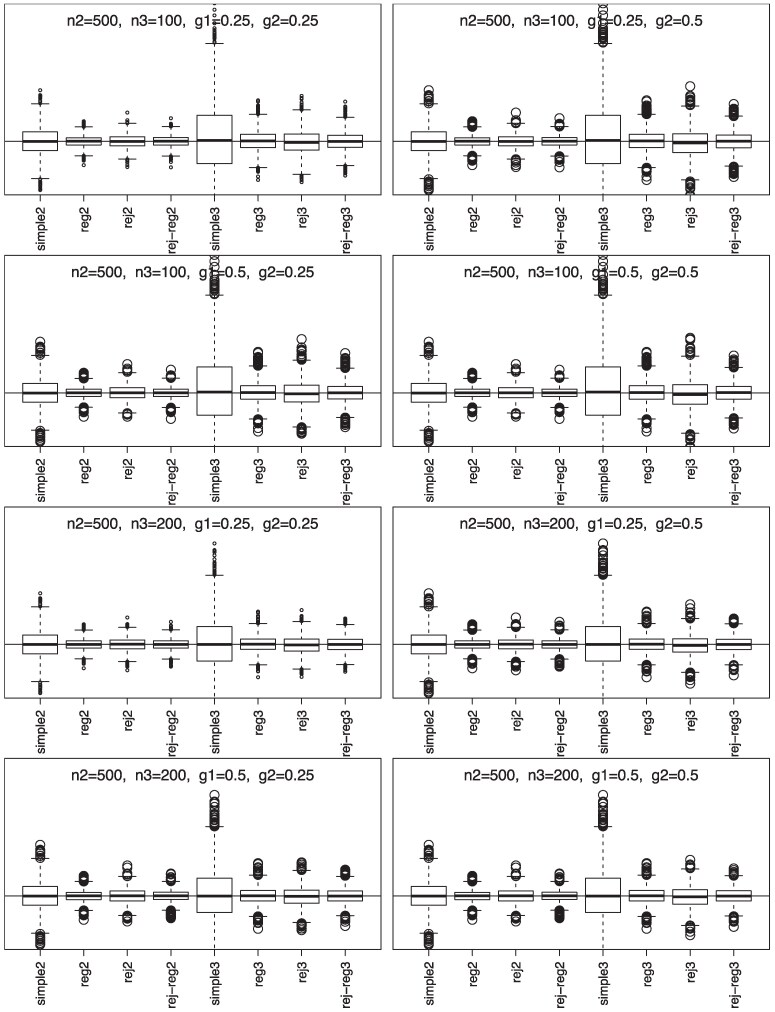
Simulation results for simple estimators and regression estimators under two/three-phase w/o rejective sampling with $n_{\mathrm{II}}=500.$  *Note*. n2 is $E(n_{\mathrm{II}})$, n3 is $E(n_{\mathrm{III}})$, g1 is $\gamma_{1}^{2}$, and g2 is $\gamma_{2}^{2}$; simple2 and reg2 are ${\bar{y}}_{\mathrm{II}}$ and ${\bar{y}}_{\mathrm{II},\mathrm{reg}}$ without rejective sampling; rej2 and rej-reg2 are ${\bar{y}}_{\mathrm{II}}$ and ${\bar{y}}_{\mathrm{II},\mathrm{reg}}$ with rejective sampling; simple3 and reg3 are ${\bar{y}}_{\mathrm{III}}$ and ${\bar{y}}_{\mathrm{III},\mathrm{reg}}$ without rejective sampling; rej3 and rej-reg3 are ${\bar{y}}_{\mathrm{III}}$ and ${\bar{y}}_{\mathrm{III},\mathrm{reg}}$ with rejective sampling.

**Figure 2.**
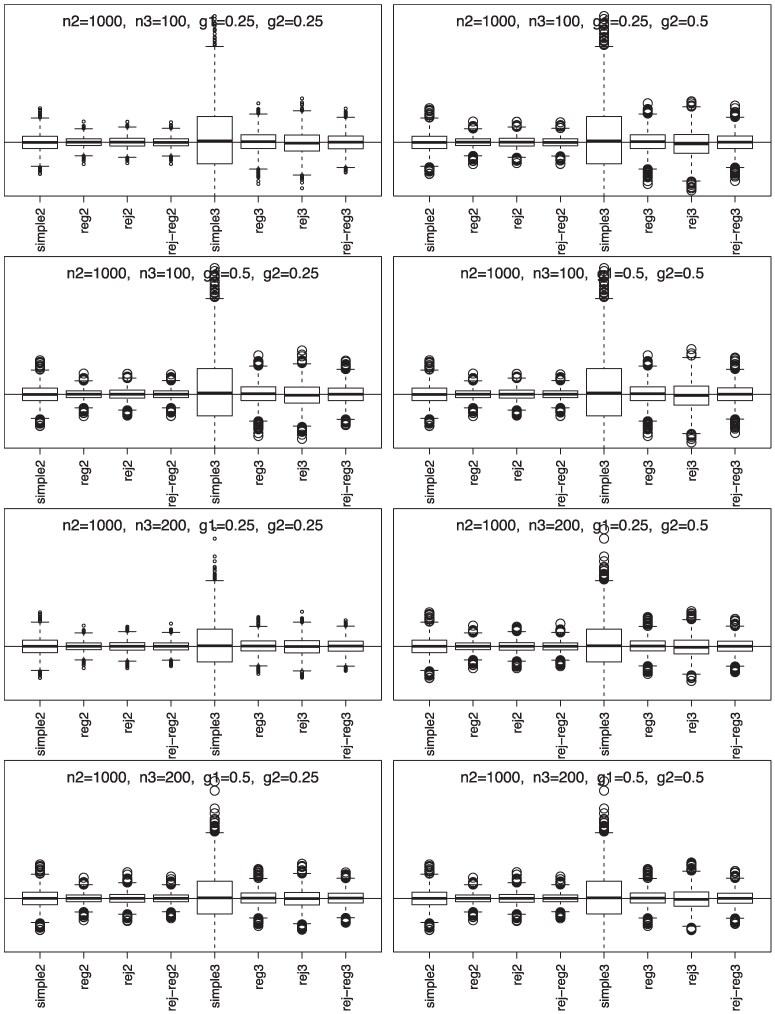
Simulation results for simple estimators and regression estimators under two/three-phase w/o rejective sampling with $n_{\mathrm{II}}=1,000.$  *Note*. n2 is $E(n_{\mathrm{II}})$, n3 is $E(n_{\mathrm{III}})$, g1 is $\gamma_{1}^{2}$, and g2 is $\gamma_{2}^{2}$; simple2 and reg2 are ${\bar{y}}_{\mathrm{II}}$ and ${\bar{y}}_{\mathrm{II},\mathrm{reg}}$ without rejective sampling; rej2 and rej-reg2 are ${\bar{y}}_{\mathrm{II}}$ and ${\bar{y}}_{\mathrm{II},\mathrm{reg}}$ with rejective sampling; simple3 and reg3 are ${\bar{y}}_{\mathrm{III}}$ and ${\bar{y}}_{\mathrm{III},\mathrm{reg}}$ without rejective sampling; rej3 and rej-reg3 are ${\bar{y}}_{\mathrm{III}}$ and ${\bar{y}}_{\mathrm{III},\mathrm{reg}}$ with rejective sampling.

**Figure 3.**
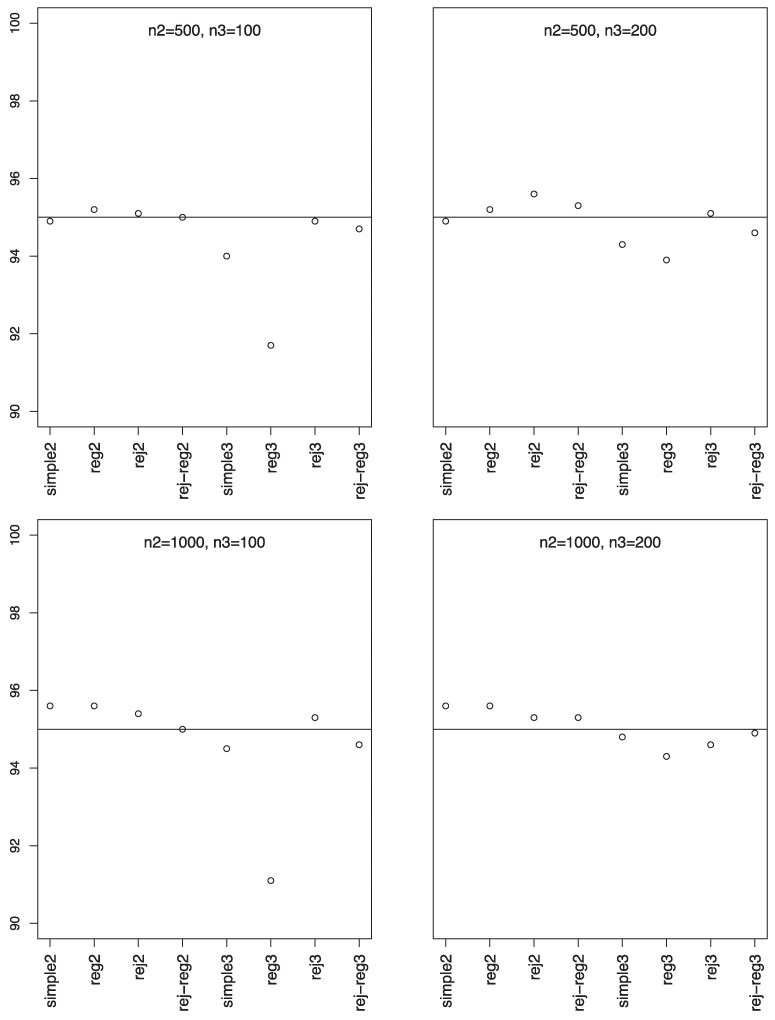
Coverage rates. *Note*. n2 is $E(n_{\mathrm{II}})$, n3 is $E(n_{\mathrm{III}})$, simple2 and reg2 are ${\bar{y}}_{\mathrm{II}}$ and ${\bar{y}}_{\mathrm{II},\mathrm{reg}}$ without rejective sampling; rej2 and rej-reg2 are ${\bar{y}}_{\mathrm{II}}$ and ${\bar{y}}_{\mathrm{II},\mathrm{reg}}$ with rejective sampling; simple3 and reg3 are ${\bar{y}}_{\mathrm{III}}$ and ${\bar{y}}_{\mathrm{III},\mathrm{reg}}$ without rejective sampling; rej3 and rej-reg3 are ${\bar{y}}_{\mathrm{III}}$ and ${\bar{y}}_{\mathrm{III},\mathrm{reg}}$ with rejective sampling.

Simulation results, summarized in Figures 1 and 2, show that phase-II estimators are more efficient due to more information collection, but phase-III designs might provide greater cost-effectiveness. For instance, phase-II rejective regression estimators with an average sample size of $n_{\mathrm{II}}=500$ had a variance of 5.4, compared with phase-III estimators with a smaller average sample size of $n_{\mathrm{III}}=100$ but a higher variance of 15.9. This implies that multiple phases can achieve desired variances with smaller samples. The efficiency of rejective regression estimators improves with tighter constraints $\gamma_{1}^{2}$ and $\gamma_{2}^{2}$ , especially $\gamma_{2}^{2}$ . Increasing the phase-II sample size showed minimal impact on the efficiency of phase-III estimator. The coverage rates of the confidence intervals align well with the nominal level, confirming our results on the asymptotic distributions (Figure 3).

## Supplementary Material

qkaf002_Supplementary_Data

## Data Availability

The data that support the findings of this study are openly available at https://r-survey.r-forge.r-project.org/survey/html/api.html.
