# Supplementary material for: Two-phase rejective sampling and its asymptotic properties
Source: J R Stat Soc Series B Stat Methodol. 2025 Feb 10;87(4):957–77. doi: 10.1093/jrsssb/qkaf002 (PMC12355938; doi:10.1093/jrsssb/qkaf002)
Supplement: qkaf002_Supplementary_Data [file qkaf002_supplementary_data.pdf]

Supplementary material

Section S1 describes sequential rejective sampling with tiers of covariates. Section S2 presents multi-phase rejective sampling. Section S3 provides all proofs.

S1 Sequential rejective sampling with tiers of covariates

Partition covariate x into K tiers of decreasing importance, denoted by $(x[1],\ldots,x[K])$, each $x[k]$ having dimension $p[k]$. Let $x[\overline{k}]=(x[1],\ldots,x[k])$. It is convenient to apply the block-wise Gram–Schmidt orthogonalization to the covariates: construct $g=(g[1],\ldots,g[K])$ from $g[1]=x[1]$, and

$$g[k]=x[k]-V_{x[k]x[\overline{k-1}],\mathrm{I}}\left(V_{x[\overline{k-1}]x[\overline{k-1}],\mathrm{I}}\right)^{-1}x[\overline{k-1}]$$

for $k=2,\ldots,K$. We assume $\mathrm{cov}(\bar{g}_{\mathrm{II}}[k],\bar{g}_{\mathrm{II}}[l]\mid\mathcal{A})=o_P(n_{\mathrm{II}}^{-1})$ a.s. for $k\neq l$ under suitable regularity conditions. See the discussion of Assumption 2(vii) in the main paper. For each tier k, we can impose a threshold $\omega_k^{-1}\gamma^2$, where γ^2 is a positive constant and ω_k is the weight for tier k, with higher ω_k indicating a stricter threshold.

We define two-phase sequential rejective sampling (TPSRS) as follows.

Definition S1 (TPSRS with general sampling) *TPSRS with general sampling consists of two steps:*

Step 1. *Select a phase-I sample $\mathcal{A}$ by a general π sampling with the inclusion probability $\pi_{\mathrm{I}i}$. For $i\in\mathcal{A}$, record x_i.*

Step 2. *Treat the phase-I sample $\mathcal{A}$ as the population and select a phase-II sample $\mathcal{B}$ by a general π sampling with the conditional inclusion probability $\pi_{\mathrm{II}i\mid\mathcal{A}}$ given unit i is in the phase-*I *sample. Accept the phase-II sample if*

$$Q_{\mathrm{I}}[k]=(\bar{g}_{\mathrm{II}}[k]-\bar{g}_{\mathrm{I}}[k])^{\mathrm{T}}V_{g[k]g[k],\mathrm{I}}^{-1}(\bar{g}_{\mathrm{II}}[k]-\bar{g}_{\mathrm{I}}[k])<\omega_k^{-1}\gamma^2,\ (1\leq k\leq K), \tag{S1}$$

done sequentially from tier 1 *to K. For $i\in\mathcal{B}$, record y_i.*

Remark S2 *In a weighted rejective sampling, we accept the phase-II sample if*

$$\sum_{k=1}^{K}\omega_k Q_{\mathrm{I}}[k]<K\gamma^2, \tag{S2}$$

where the ω_k's may be different from the ones in the sequential rejective sampling as in (S1).

Sequential rejective sampling, unlike weighted rejective sampling, ensures balance across all tiers, as it requires each tier to individually meet its threshold. Weighted rejective sampling is less stringent, allowing some tiers to be poorly balanced if others are well balanced. Zhong and Rubin (2024) studied

the admissibility of different rerandomization criteria. The limiting distributions of $\bar{y}_{\mathrm{II}}$ *and* $\bar{y}_{\mathrm{II,reg}}$ *under weighted rejective sampling (S2) are more complicated compared with the TPSRS in Definition S1 (Lu et al., 2023).*

To study the limiting properties of $\bar{y}_{\mathrm{II}}$, let β_{gy} be the probability limit of $V_{gg,\mathrm{I}}^{-1}V_{gy,\mathrm{I}}$. By the Gram–Schmidt orthogonalization, $V_{gg,\mathrm{I}}$ is a diagonal matrix with the kth block diagonal component $V_{g[k]g[k],\mathrm{I}}$. We decompose β_{gy} as $\beta_{gy} = (\beta_{g[1]y}, \ldots, \beta_{g[K]y})$ with $\beta_{g[k]y}$ being the probability of $V_{g[k]g[k],\mathrm{I}}^{-1}V_{g[k]y,\mathrm{I}}$. The decomposition of $\bar{y}_{\mathrm{II}} - \bar{y}_0$ then becomes

$$\begin{aligned}\bar{y}_{\mathrm{II}} - \bar{y}_0 &= (\bar{g}_{\mathrm{II}} - \bar{g}_{\mathrm{I}})^{\mathrm{T}}\beta_{gy} + \{\bar{y}_{\mathrm{II}} - \bar{y}_{\mathrm{I}} - (\bar{g}_{\mathrm{II}} - \bar{g}_{\mathrm{I}})^{\mathrm{T}}\beta_{gy}\} + \bar{y}_{\mathrm{I}} - \bar{y}_0 \\ &= \sum_{k=1}^{K}(\bar{g}_{\mathrm{II}}[k] - \bar{g}_{\mathrm{I}}[k])^{\mathrm{T}}\beta_{g[k]y} + \{\bar{y}_{\mathrm{II}} - \bar{y}_{\mathrm{I}} - (\bar{g}_{\mathrm{II}} - \bar{g}_{\mathrm{I}})^{\mathrm{T}}\beta_{gy}\} + \bar{y}_{\mathrm{I}} - \bar{y}_0.\end{aligned}$$

Let $D_{\mathrm{I}}[k] = V_{g[k]g[k],\mathrm{I}}^{-1/2}(\bar{g}_{\mathrm{II}}[k] - \bar{g}_{\mathrm{I}}[k])$, then $D_{\mathrm{I}}[k] \to \mathcal{N}(0, I_{p[k]})$. For any $k \neq l$, $\bar{g}_{\mathrm{II}}[k] - \bar{g}_{\mathrm{I}}[k]$ and $\bar{g}_{\mathrm{II}}[l] - \bar{g}_{\mathrm{I}}[l]$ are asymptotically independent given $\mathcal{A}$ a.s.. Therefore,

$$\sum_{k=1}^{K}(\bar{g}_{\mathrm{II}}[k] - \bar{g}_{\mathrm{I}}[k])^{\mathrm{T}}\beta_{g[k]y} \mid \left(D_{\mathrm{I}}^{\mathrm{T}}[k]D_{\mathrm{I}}[k] < \omega_k^{-1}\gamma^2 : 1 \leq k \leq K\right)$$

has the same limiting distribution of

$$\sum_{k=1}^{K}(\bar{g}_{\mathrm{II}}[k] - \bar{g}_{\mathrm{I}}[k])^{\mathrm{T}}\beta_{g[k]y} \mid \left(D_{\mathrm{I}}^{\mathrm{T}}[k]D_{\mathrm{I}}[k] < \omega_k^{-1}\gamma^2\right).$$

Theorem S6 below describes the limiting distributions of $\bar{y}_{\mathrm{II}}$ and $\bar{y}_{\mathrm{II,reg}}$ under TPSRS with general sampling.

Theorem S6 *Suppose Assumption 2 holds. Under TPSRS in Definition S1,* $\bar{y}_{\mathrm{II}}$ *follows the limiting distribution:*

$$n_{\mathrm{II}}^{1/2}(\bar{y}_{\mathrm{II}} - \bar{y}) \mid \left(D_{\mathrm{I}}^{\mathrm{T}}[k]D_{\mathrm{I}}[k] < \omega_k^{-1}\gamma^2 : 1 \leq k \leq K\right) \to \sum_{k=1}^{K} V_1^{1/2}[k]L_{p[k],\omega_k^{-1}\gamma^2} + V_2^{1/2}Z_1 + V_3^{1/2}Z_2, \quad \text{(S3)}$$

where $V_1[k] = \lim_{n_{\mathrm{II}}\to\infty} n_{\mathrm{II}}\beta_{g[k]y}^{\mathrm{T}}E(V_{g[k]g[k],\mathrm{I}} \mid \mathcal{F})\beta_{g[k]y}$, V_2 *and* V_3 *are defined in (22) and (23), respectively,* Z_1 *and* Z_2 *are independent standard normal variables, and* $(L_{p[k],\omega_k^{-1}\gamma^2}, Z_1, Z_2)$ *are jointly independent. Moreover,*

$$n_{\mathrm{II}}^{1/2}(\bar{y}_{\mathrm{II,reg}} - \bar{y}_0) \mid \left(D_{\mathrm{I}}^{\mathrm{T}}[k]D_{\mathrm{I}}[k] < \omega_k^{-1}\gamma^2 : 1 \leq k \leq K\right) \to V_2^{1/2}Z_1 + V_3^{1/2}Z_2.$$

S2 Multi-phase rejective sampling

S2.1 Notation

We extend our theory to multi-phase rejective sampling. To simplify the presentation, we discuss three-phase sampling below. Let $\pi_{\mathrm{I}i}$, $\pi_{\mathrm{II}i|\mathcal{A}}$, and $\pi_{\mathrm{III}i|\mathcal{B}}$ represent the probabilities of a unit being included in the phase-I, phase-II, and phase-III samples, with each phase indexed by $\mathcal{A}$, $\mathcal{B}$ and $\mathcal{C}$, respectively. The combined probability for phase-III is $\pi^*_{\mathrm{III}i} = \pi_{\mathrm{I}i}\pi_{\mathrm{II}i|\mathcal{A}}\pi_{\mathrm{III}i|\mathcal{B}}$. Let n_{I}, n_{II}, and n_{III} be the sampling sizes of the phase-I sample, the phase-II sample, and the phase-III sample, respectively.

For the finite population mean $\bar{u}_0$, the phase-I, phase-II, and phase-III estimators are (14), (15), and

$$\bar{u}_{\mathrm{III}} = \frac{1}{\sum_{i\in\mathcal{C}}(\pi^*_{\mathrm{III}i})^{-1}} \sum_{i\in\mathcal{C}} \frac{u_i}{\pi^*_{\mathrm{III}i}}, \tag{S4}$$

respectively. The design variance for the phase-III estimator involves conditional second-order inclusion probabilities $\pi_{\mathrm{III}ij|\mathcal{B}}$. By Taylor expansion and ignoring the small order terms, the design covariance of $\bar{u}_{\mathrm{III}\pi}$ and $\bar{v}_{\mathrm{III}\pi}$ given the phase-II sample is

$$V_{uv,\mathrm{II}} = \mathrm{cov}\left(\bar{u}_{\mathrm{III}\pi}, \bar{v}_{\mathrm{III}\pi} \mid \mathcal{B}, \mathcal{A}, \mathcal{F}\right) = \frac{1}{N^2}\sum_{i\in\mathcal{B}}\sum_{j\in\mathcal{B}} \frac{\pi_{\mathrm{III}ij|\mathcal{B}} - \pi_{\mathrm{III}i|\mathcal{B}}\pi_{\mathrm{III}j|\mathcal{B}}}{\pi^*_{\mathrm{III}i}\pi^*_{\mathrm{III}j}}(u_i - \bar{u}_{\mathrm{II}})(v_j - \bar{v}_{\mathrm{II}})^{\mathrm{T}}. \tag{S5}$$

S2.2 Three-phase rejective sampling

We define a three-phase rejective sampling method with p_1-dimensional phase-I covariate x and p_2-dimensional phase-II covariate z as follows.

Definition S2 (Three-phase rejective sampling with general sampling) *Three-phase rejective sampling with general sampling consists of three steps:*

Step 1. *Select a phase-*I *sample* $\mathcal{A}$ *by a general* π *sampling with the inclusion probability* $\pi_{\mathrm{I}i}$. *For* $i \in \mathcal{A}$, *record* x_i.

Step 2. *Select a phase-*II *sample* $\mathcal{B}$ *by a general* π *sampling with the conditional inclusion probability* $\pi_{\mathrm{II}i|\mathcal{A}}$ *given unit* i *is in the phase-*I *sample. Accept the phase-*II *sample if*

$$Q_{x,\mathrm{I}} = (\bar{x}_{\mathrm{II}} - \bar{x}_{\mathrm{I}})^{\mathrm{T}}\, V^{-1}_{xx,\mathrm{I}}\, (\bar{x}_{\mathrm{II}} - \bar{x}_{\mathrm{I}}) < \gamma_1^2,$$

where $\gamma_1^2 > 0$, *and* $V_{xx,\mathrm{I}}$ *is the positive definite design variance of* $\bar{x}_{\mathrm{II}} - \bar{x}_{\mathrm{I}}$ *given the phase-*I *sample. For* $i \in \mathcal{B}$, *record* z_i. *It is convenient to apply the Gram–Schmidt orthogonalization to* z_i, *and construct a phase-*II *covariate* $a_i = z_i - \bar{z}_{\mathrm{II}} - (x_i - \bar{x}_{\mathrm{II}})^{\mathrm{T}}\hat{\beta}_{zx,\mathrm{II}}$, *where*

$$\hat{\beta}_{zx,\mathrm{II}} = \left\{\sum_{i\in\mathcal{B}} \frac{(x_i - \bar{x}_{\mathrm{II}})^{\otimes 2}}{\pi^*_{\mathrm{II}i}}\right\}^{-1} \sum_{i\in\mathcal{B}} \frac{(x_i - \bar{x}_{\mathrm{II}})(z_i - \bar{z}_{\mathrm{II}})^{\mathrm{T}}}{\pi^*_{\mathrm{II}i}}. \tag{S6}$$

*Let $c = (x^{\mathrm{T}}, a^{\mathrm{T}})^{\mathrm{T}}$ represent the combined vector of phase-*II *covariates, where a can be seen as the part of z that is both correlated with y and orthogonal to x, representing the additional information beyond what is available from phase* I.

Step 3. *Select a phase-*III *rejective sample $\mathcal{C}$ by a general π sampling with the conditional inclusion probability $\pi_{\mathrm{III}i|\mathcal{B}}$ given unit i is in the phase-*II *sample. Accept the phase-*III *sample if*

$$Q_{c,\mathrm{II}} = (\bar{c}_{\mathrm{III}} - \bar{c}_{\mathrm{II}})^{\mathrm{T}} V_{cc,\mathrm{II}}^{-1} (\bar{c}_{\mathrm{III}} - \bar{c}_{\mathrm{II}}) < \gamma_2^2,$$

*where $\gamma_2^2 > 0$, and $V_{cc,\mathrm{II}}$ is the positive definite design variance of $\bar{c}_{\mathrm{III}} - \bar{c}_{\mathrm{II}}$ given the phase-*II *sample. For $i \in \mathcal{C}$, record y_i.*

In Step 3, balance can be directly controlled on $(x^{\mathrm{T}}, z^{\mathrm{T}})^{\mathrm{T}}$ instead of c, because $\bar{c}_{\mathrm{III}} - \bar{c}_{\mathrm{II}}$ is a linear transformation of $\bar{x}_{\mathrm{III}} - \bar{x}_{\mathrm{II}}$ and $\bar{z}_{\mathrm{III}} - \bar{z}_{\mathrm{II}}$ given $\hat{\beta}_{zx,\mathrm{II}}$ and the Mahalanobis distance is invariant to linear transformations.

For the population mean $\bar{y}_0$, the π^* estimator based on the phase-III rejective sample is

$$\bar{y}_{\mathrm{III}} = \frac{1}{\sum_{i\in\mathcal{C}} (\pi_{\mathrm{III}i}^*)^{-1}} \sum_{i\in\mathcal{C}} \frac{y_i}{\pi_{\mathrm{III}i}^*}.$$

To derive the asymptotic properties of $\bar{y}_{\mathrm{III}}$, we need the following regularity condition.

Assumption S1 *The phase-*III *estimator (S4) satisfies*

$$\mathrm{var}(\hat{u}_{\mathrm{III}} \mid \mathcal{B}, \mathcal{A}, \mathcal{F})^{-1/2} (\hat{u}_{\mathrm{III}} - \bar{u}_{\mathrm{II}}) \mid \mathcal{B}, \mathcal{A}, \mathcal{F} \to \mathcal{N}(0,1) \text{ a.s.},$$

with $\mathrm{var}(n_{\mathrm{III}}^{1/2} \hat{u}_{\mathrm{III}} \mid \mathcal{B}, \mathcal{A}, \mathcal{F}) = O_P(1)$, where u represents components of either x or y.

*The sequence of phase-*II *selection probabilities are bounded by $K_{\mathrm{III,L}} < n_{\mathrm{III}}^{-1} n_{\mathrm{II}} \pi_{\mathrm{III}i} < K_{\mathrm{III,U}}$ for all i, for some positive $K_{\mathrm{III,L}} > 0$ and $K_{\mathrm{III,U}} > 0$. Assume that the design is such that*

$$\lim_{N\to\infty} \sum_{i\in\mathcal{C}} \pi_{\mathrm{III}i}^{-1} (1, c_i^{\mathrm{T}}, y_i, y_i^2)^{\mathrm{T}} (1, c_i^{\mathrm{T}}, y_i, y_i^2) = M_{\mathrm{III}} \text{ a.s.},$$

where M_{III} is a matrix of constants. Moreover, $\mathrm{cov}(\bar{c}_{\mathrm{III}} - \bar{c}_{\mathrm{II}}, \bar{e}_{\mathrm{III}} - \bar{e}_{\mathrm{II}} \mid \mathcal{B}, \mathcal{A}, \mathcal{F}) = o_P(n_{\mathrm{III}}^{-1})$.

Theorem S7 *Suppose Assumptions 2 and S1 hold. Under three-phase rejective sampling in Definition S2, $\bar{y}_{\mathrm{III}}$ follows the limiting distribution:*

$$n_{\mathrm{III}}^{1/2} (\bar{y}_{\mathrm{III}} - \bar{y}_0) \mid (Q_{z,\mathrm{I}} < \gamma_1^2, Q_{c,\mathrm{II}} < \gamma_2^2) \to V_{\mathrm{III},1}^{1/2} L_{p_1+p_2,\gamma_2^2} + V_{\mathrm{III},2}^{1/2} Z_1 + V_{\mathrm{III},3}^{1/2} L_{p_1,\gamma_1^2} + V_{\mathrm{III},4}^{1/2} Z_2 + V_{\mathrm{III},5}^{1/2} Z_3, \quad \text{(S7)}$$

where

$$V_{\mathrm{III},1} = \lim_{N\to\infty} n_{\mathrm{III}} \beta_{yc,0}^{\mathrm{T}} E\left(V_{cc,\mathrm{II}} \mid \mathcal{F}\right) \beta_{yc,0}, \qquad V_{\mathrm{III},2} = \lim_{N\to\infty} n_{\mathrm{III}} E\left(V_{e_{yc}e_{yc},\mathrm{II}} \mid \mathcal{F}\right),$$

$$V_{\mathrm{III},3} = \lim_{N\to\infty} n_{\mathrm{III}} \beta_{yx,0}^{\mathrm{T}} E(V_{xx,\mathrm{I}} \mid \mathcal{F}) \beta_{yx,0}, \qquad V_{\mathrm{III},4} = \lim_{N\to\infty} n_{\mathrm{III}} E\left(V_{e_{yx}e_{yx},\mathrm{I}} \mid \mathcal{F}\right),$$
$$V_{\mathrm{III},5} = \lim_{N\to\infty} n_{\mathrm{III}} V_{yy,0}, \qquad e_{uv} = u - v^{\mathrm{T}} \beta_{uv,0}, \tag{S8}$$

Z_1, Z_2, and Z_3 are standard normal variables, and $(Z_1, Z_2, Z_3, L_{p_1,\gamma_1^2}, L_{p_1+p_2,\gamma_2^2})$ are jointly independent.

S2.3 Regression estimator

Integrating the design and analysis strategies, the three-phase regression estimator of $\bar{y}_0$ is

$$\bar{y}_{\mathrm{III,reg}} = \bar{y}_{\mathrm{III}} + \begin{pmatrix} \bar{x}_{\mathrm{I}} - \bar{x}_{\mathrm{III}} \\ \bar{a}_{\mathrm{II}} - \bar{a}_{\mathrm{III}} \end{pmatrix}^{\mathrm{T}} \hat{\beta}_{yc,\mathrm{III}}, \tag{S9}$$

where $\bar{a}_{\mathrm{II}} = 0$ by our construction of a_i, and

$$\hat{\beta}_{yc,\mathrm{III}} = \left\{ \sum_{i\in\mathcal{C}} \frac{(c_i - \bar{c}_{\mathrm{III}})(c_i - \bar{c}_{\mathrm{III}})^{\mathrm{T}}}{\pi_{\mathrm{III}i}^*} \right\}^{-1} \sum_{i\in\mathcal{C}} \frac{(c_i - \bar{c}_{\mathrm{III}})(y_i - \bar{y}_{\mathrm{III}})}{\pi_{\mathrm{III}i}^*}. \tag{S10}$$

The regression estimator exhibits the same asymptotic behavior, irrespective of the use of rejective sampling.

Theorem S8 *Under Assumptions 2 and S1, under three-phase rejective sampling in Definition S2, the regression estimator $\bar{y}_{\mathrm{III,reg}}$ in (S9) has the following limiting distribution:*

$$n_{\mathrm{III}}^{1/2}(\bar{y}_{\mathrm{III,reg}} - \bar{y}_0) \mid \mathcal{F} \to \mathcal{N}(0, V_{\mathrm{III,reg}}),$$

a.s. *for all sequences of finite populations, where*

$$V_{\mathrm{III,reg}} = \lim_{N\to\infty} n_{\mathrm{III}} \left\{ V_{yy,0} + E\left(V_{e_{yx}e_{yx},\mathrm{I}} \mid \mathcal{F}\right) + E\left(V_{e_{yc}e_{yc},\mathrm{II}} \mid \mathcal{F}\right) \right\},$$

and $V_{uv,0}$, $V_{uv,\mathrm{I}}$ and $V_{uv,\mathrm{II}}$ are defined in (16), (17) and (S5), respectively.

Thus, the limiting distribution of $n_{\mathrm{III}}^{1/2}(\bar{y}_{\mathrm{III,reg}} - \bar{y}_0)$ remains unchanged with or without rejective procedure. Combining the results from Theorems S7 and S8 suggests that utilizing auxiliary variables in both the design stage (via rejective sampling) and analysis stage (via regression) can improve estimation efficiency.

S2.4 Inference: variance estimators and confidence intervals

We derive the asymptotic design variance formula. Let $\hat{\beta}_{uv,\mathrm{III}}$ be (S10) with y_i and c_i being u_i and v_i, $\hat{e}_{uv,i} = u_i - v_i^{\mathrm{T}} \hat{\beta}_{uv,\mathrm{III}}$, and $\hat{e}_{uv,\mathrm{III}}$ be (S4) with u_i being $\hat{e}_{uv,i}$. We estimate $V_{yy,0}$, $V_{e_{yx}e_{yx},\mathrm{I}}$ and

$V_{e_{yc}e_{yc},\mathrm{II}}$ by

$$\begin{aligned}
\hat{V}_{yy,0} &= \frac{1}{N^2}\sum_{i\in\mathcal{A}_{\mathrm{III}}}\sum_{j\in\mathcal{A}_{\mathrm{III}}}\frac{\pi_{\mathrm{I}ij}-\pi_{\mathrm{I}i}\pi_{\mathrm{I}j}}{\pi_{\mathrm{I}i}\pi_{\mathrm{I}j}}\frac{(y_i-\bar{y}_{\mathrm{III}})(y_j-\bar{y}_{\mathrm{III}})^{\mathrm{T}}}{\pi_{\mathrm{I}ij}\pi_{\mathrm{II}ij\mid\mathcal{A}}\pi_{\mathrm{III}ij\mid\mathcal{B}}},\\
\hat{V}_{e_{yx}e_{yx},\mathrm{I}} &= \frac{1}{N^2}\sum_{i\in\mathcal{A}_{\mathrm{III}}}\sum_{j\in\mathcal{A}_{\mathrm{III}}}\frac{\pi_{\mathrm{II}ij\mid\mathcal{A}}-\pi_{\mathrm{II}i\mid\mathcal{A}}\pi_{\mathrm{II}j\mid\mathcal{A}}}{\pi^*_{\mathrm{II}i}\pi^*_{\mathrm{II}j}}\frac{(\hat{e}_{yx,i}-\hat{e}_{yx,\mathrm{III}})(\hat{e}_{yx,j}-\hat{e}_{yx,\mathrm{III}})^{\mathrm{T}}}{\pi_{\mathrm{II}ij\mid\mathcal{A}}\pi_{\mathrm{III}ij\mid\mathcal{A}}},\\
\hat{V}_{e_{yc}e_{yc},\mathrm{II}} &= \frac{1}{N^2}\sum_{i\in\mathcal{A}_{\mathrm{III}}}\sum_{j\in\mathcal{A}_{\mathrm{III}}}\frac{\pi_{\mathrm{III}ij\mid\mathcal{B}}-\pi_{\mathrm{III}i\mid\mathcal{B}}\pi_{\mathrm{III}j\mid\mathcal{B}}}{\pi^*_{\mathrm{III}i}\pi^*_{\mathrm{III}j}}\frac{(\hat{e}_{yc,i}-\hat{e}_{yc,\mathrm{III}})(\hat{e}_{yc,j}-\hat{e}_{yc,\mathrm{III}})^{\mathrm{T}}}{\pi_{\mathrm{III}ij\mid\mathcal{B}}},
\end{aligned}$$

respectively. Then, the variance estimator for $\bar{y}_{\mathrm{III}}$ is

$$\hat{V}_{\mathrm{III}} = \left(\hat{\beta}^{\mathrm{T}}_{yc,\mathrm{III}}V_{cc,\mathrm{II}}\hat{\beta}_{yc,\mathrm{III}}\right)v_{p_1+p_2,\gamma_2^2} + \left(\hat{\beta}^{\mathrm{T}}_{yx,\mathrm{III}}V_{xx,\mathrm{I}}\hat{\beta}_{yx,\mathrm{III}}\right)v_{p_1,\gamma_1^2} + \hat{V}_{e_{yc}e_{yc},\mathrm{II}} + \hat{V}_{e_{yx}e_{yx},\mathrm{I}} + \hat{V}_{yy,0}.$$

The variance estimator for $\bar{y}_{\mathrm{III,reg}}$ is $\hat{V}_{\mathrm{III,reg}} = \hat{V}_{yy,0} + \hat{V}_{e_{yx}e_{yx},\mathrm{I}} + \hat{V}_{e_{yc}e_{yc},\mathrm{II}}$.

We can construct the asymptotic $(1-\alpha)$ confidence interval for $\bar{y}_0$ based on $\bar{y}_{\mathrm{III}}$ as

$$\left(\bar{y}_{\mathrm{III}} - n_{\mathrm{III}}^{-1/2}\nu_{1-\alpha/2}(\hat{V}_{\mathrm{III},1},\cdots,\hat{V}_{\mathrm{III},5}),\ \bar{y}_{\mathrm{III}} - n_{\mathrm{III}}^{-1/2}\nu_{\alpha/2}(\hat{V}_{\mathrm{III},1},\cdots,\hat{V}_{\mathrm{III},5})\right),$$

where $\nu_\alpha(V_{\mathrm{III},1},\cdots,V_{\mathrm{III},5})$ as the 100αth quantile of the distribution of $V_{\mathrm{III},1}^{1/2}L_{p_1+p_2,\gamma_2^2} + V_{\mathrm{III},2}^{1/2}Z_1 + V_{\mathrm{III},3}^{1/2}L_{p_1,\gamma_1^2} + V_{\mathrm{III},4}^{1/2}Z_2 + V_{\mathrm{III},5}^{1/2}Z_3$, and the counterpart based on $\bar{y}_{\mathrm{II,reg}}$ as

$$\left(\bar{y}_{\mathrm{III,reg}} - \hat{V}_{\mathrm{III,reg}}^{1/2}z_{1-\alpha/2},\ \bar{y}_{\mathrm{III}} - \hat{V}_{\mathrm{III,reg}}^{1/2}z_{\alpha/2}\right).$$

S3 Proofs

S3.1 Useful lemmas

We state some useful lemmas for two-phase simple random sampling.

Lemma S1 $E(\bar{u}_{\mathrm{II}} \mid \mathcal{A},\mathcal{F}) = \bar{u}_{\mathrm{I}}$ *and* $E(\bar{u}_{\mathrm{I}} \mid \mathcal{F}) = \bar{u}_0$.

Lemma S2 $\mathrm{cov}(\bar{u}_{\mathrm{II}},\bar{v}_{\mathrm{II}} \mid \mathcal{A},\mathcal{F}) = \left(n_{\mathrm{II}}^{-1} - n_{\mathrm{I}}^{-1}\right)V_{uv,\mathrm{I}}$ *and* $\mathrm{cov}(\bar{u}_{\mathrm{I}},\bar{v}_{\mathrm{I}} \mid \mathcal{F}) = \left(n_{\mathrm{I}}^{-1} - N^{-1}\right)V_{uv,0}$.

Lemma S3 $E(V_{uv,\mathrm{II}} \mid \mathcal{A},\mathcal{F}) = V_{uv,\mathrm{I}}$ *and* $E(V_{uv,\mathrm{I}} \mid \mathcal{F}) = V_{uv,0}$.

Lemmas S2 and S3 are standard textbook results (e.g., Fuller, 2009a); therefore, we omit their proofs.

For a sequence of finite populations, the variability of the estimators comes from the sampling design. In the following proofs for the asymptotic design properties, the asymptotic design variance and covariance (the limits of the design variance and covariances), denoted by a.var and a.cov, respectively, are relevant.

S3.2 Proof of Lemma 1

Recall the following definitions $T_1 = n_{\mathrm{II}}^{1/2}(\bar{x}_{\mathrm{II}} - \bar{x}_{\mathrm{I}})^{\mathrm{T}}\beta_0$, $T_2 = n_{\mathrm{II}}^{1/2}\{\bar{y}_{\mathrm{II}} - \bar{y}_{\mathrm{I}} - (\bar{x}_{\mathrm{II}} - \bar{x}_{\mathrm{I}})^{\mathrm{T}}\beta_0\}$, and $T_3 = n_{\mathrm{II}}^{1/2}(\bar{y}_{\mathrm{I}} - \bar{y}_0)$. The asymptotic normality follows by Assumption 1; see, e.g., Chapter 1 in Fuller (2009a).

Because $\mathcal{B}$ is a simple random sample from $\mathcal{A}$, and $\mathcal{A}$ is a simple random sample from $\mathcal{F}$, by Lemma S1, we have

$$E(T_1 \mid \mathcal{A}, \mathcal{F}) = 0,\ E(T_2 \mid \mathcal{A}, \mathcal{F}) = 0,\ E(T_3 \mid \mathcal{F}) = 0, \tag{S11}$$

and therefore $E(T_k \mid \mathcal{F}) = 0$, for $k = 1, 2, 3$.

We then show the asymptotic variance formulas. First, the asymptotic design variance of T_1 given $\mathcal{F}$ is

$$\begin{aligned}
\mathrm{a.var}(T_1 \mid \mathcal{F}_N) &\equiv \lim_{N\to\infty} \mathrm{var}(T_1 \mid \mathcal{F}) \\
&= \lim_{N\to\infty} [\mathrm{var}\{E(T_1 \mid \mathcal{A}, \mathcal{F}) \mid \mathcal{F}\} + E\{\mathrm{var}(T_1 \mid \mathcal{A}, \mathcal{F}) \mid \mathcal{F}\}] \\
&= \lim_{N\to\infty} \left[0 + E\left\{\left(1 - \frac{n_{\mathrm{II}}}{n_{\mathrm{I}}}\right)\beta_0^{\mathrm{T}} V_{xx,\mathrm{I}}\beta_0 \mid \mathcal{F}\right\}\right] \\
&= \lim_{N\to\infty} \left(1 - \frac{n_{\mathrm{II}}}{n_{\mathrm{I}}}\right)\beta_0^{\mathrm{T}} V_{xx,0}\beta_0 \\
&= \lim_{N\to\infty} \left(1 - \frac{n_{\mathrm{II}}}{n_{\mathrm{I}}}\right) V_{yx,0}V_{xx,0}^{-1}V_{xy,0} \\
&= (1 - f_{\mathrm{II,I}})\,\sigma_{yx}\sigma_{xx}^{-1}\sigma_{xy}, \text{ a.s.,}
\end{aligned}$$

where the second equality follows by (S11) and Lemma S2, and the third equality follows by Lemma S3. Second, by writing $T_2 = n_{\mathrm{II}}^{1/2}(\bar{e}_{\mathrm{II}} - \bar{e}_{\mathrm{I}}) + o_P(1)$, the asymptotic design variance of T_2 given $\mathcal{F}$ is

$$\begin{aligned}
\mathrm{a.var}(T_2 \mid \mathcal{F}) &\equiv \lim_{N\to\infty} \mathrm{var}(T_2 \mid \mathcal{F}) \\
&= \lim_{N\to\infty} [\mathrm{var}\{E(T_2 \mid \mathcal{A}, \mathcal{F}) \mid \mathcal{F}\} + E\{\mathrm{var}(T_2 \mid \mathcal{A}, \mathcal{F}) \mid \mathcal{F}\}] \\
&= \lim_{N\to\infty} \left[0 + E\left\{\left(1 - \frac{n_{\mathrm{II}}}{n_{\mathrm{I}}}\right) V_{ee,\mathrm{I}} \mid \mathcal{F}\right\}\right] \\
&= \lim_{N\to\infty} \left(1 - \frac{n_{\mathrm{II}}}{n_{\mathrm{I}}}\right) V_{ee,0} \\
&= (1 - f_{\mathrm{II,I}})\,\sigma_{ee}, \text{ a.s.,}
\end{aligned}$$

where the second equality follows by (S11) and Lemma S2, and the third equality follows by Lemma S3. Third, the asymptotic design variance of T_3 given $\mathcal{F}$ is $\mathrm{a.var}(T_3 \mid \mathcal{F}) \equiv \lim_{N\to\infty} \mathrm{var}(T_3 \mid \mathcal{F}) = \lim_{N\to\infty}(n_{\mathrm{II}}/n_{\mathrm{I}} - n_{\mathrm{II}}/N)V_{yy,0} = f_{\mathrm{II,I}}(1 - f_{\mathrm{I},0})\sigma_{yy}$, a.s..

Finally, we show that all covariances of cross terms are zero asymptotically. We have

$$\mathrm{a.cov}(T_1, T_2 \mid \mathcal{A}, \mathcal{F}) \equiv \lim_{N\to\infty} \mathrm{cov}(T_1, T_2 \mid \mathcal{A}, \mathcal{F})$$

$$
\begin{aligned}
&= \lim_{N\to\infty} n_{\mathrm{II}}\beta_0^{\mathrm{T}}\mathrm{cov}(\bar{x}_{\mathrm{II}}-\bar{x}_{\mathrm{I}}, \bar{y}_{\mathrm{II}}-\bar{y}_{\mathrm{I}} \mid \mathcal{A},\mathcal{F}) \\
&\quad - \lim_{N\to\infty} n_{\mathrm{II}}\beta_0^{\mathrm{T}}\mathrm{cov}(\bar{x}_{\mathrm{II}}-\bar{x}_{\mathrm{I}}, \bar{x}_{\mathrm{II}}-\bar{x}_{\mathrm{I}} \mid \mathcal{A},\mathcal{F})\beta_0 \\
&= \lim_{N\to\infty}\left(1-\frac{n_{\mathrm{II}}}{n_{\mathrm{I}}}\right)(\beta_0^{\mathrm{T}}V_{xy,\mathrm{I}} - \beta_0^{\mathrm{T}}V_{xx,\mathrm{I}}\beta_0) \\
&= \lim_{N\to\infty}\left(1-\frac{n_{\mathrm{II}}}{n_{\mathrm{I}}}\right)\left(V_{yx,0}V_{xx,0}^{-1}V_{xy,\mathrm{I}} - V_{yx,0}V_{xx,0}^{-1}V_{xx,\mathrm{I}}V_{xx,0}^{-1}V_{xy,0}\right) \\
&= (1-f_{\mathrm{II},\mathrm{I}})\left(\sigma_{yx}\sigma_{xx}^{-1}\sigma_{xy} - \sigma_{yx}\sigma_{xx}\sigma_{xx}^{-1}\sigma_{xx}\sigma_{xy}\right) = 0, \text{ a.s.,} \qquad \text{(S12)}
\end{aligned}
$$

where the second equality follows by Lemma S3. Because conditional on $\mathcal{A}$ and $\mathcal{F}$, T_3 is a constant, we then have

$$
\mathrm{a.cov}(T_k, T_3 \mid \mathcal{A},\mathcal{F}) = 0, \ k=1,2. \qquad \text{(S13)}
$$

Combining (S11)–(S13), we have

$$
\begin{aligned}
\mathrm{a.cov}(T_j, T_k \mid \mathcal{F}) &\equiv \lim_{N\to\infty}\mathrm{cov}(T_j, T_k \mid \mathcal{F}) \\
&= \lim_{N\to\infty} E\{\mathrm{cov}(T_j, T_k \mid \mathcal{A},\mathcal{F}) \mid \mathcal{F}\} \\
&\quad + \lim_{N\to\infty}\mathrm{cov}\{E(T_j \mid \mathcal{A},\mathcal{F}), E(T_k \mid \mathcal{A},\mathcal{F}) \mid \mathcal{F}\} \\
&= 0, \ (j \neq k \in \{1,2,3\}), \text{ a.s..}
\end{aligned}
$$

Combining all results, we complete the proof.

S3.3 Proof of Theorem 2

To express (11) further, by the moment condition in Assumption 1, we have $n_{\mathrm{II}}^{1/2}(\bar{x}_{\mathrm{II}}-\bar{x}_{\mathrm{I}}) = O_P(1)$,

$$
\begin{aligned}
\hat{\beta}_{\mathrm{II}} - \beta_0 &= \left\{\sum_{i\in\mathcal{B}}(x_i-\bar{x}_{\mathrm{II}})(x_i-\bar{x}_{\mathrm{II}})^{\mathrm{T}}\right\}^{-1}\sum_{i\in\mathcal{B}}(x_i-\bar{x}_{\mathrm{II}})\left\{y_i-\bar{y}_{\mathrm{II}}-(x_i-\bar{x}_{\mathrm{II}})^{\mathrm{T}}\beta_0\right\} \\
&= \left\{n_{\mathrm{II}}^{-1}\sum_{i\in\mathcal{B}}(x_i-\bar{x}_{\mathrm{II}})(x_i-\bar{x}_{\mathrm{II}})^{\mathrm{T}}\right\}^{-1} n_{\mathrm{II}}^{-1}\sum_{i\in\mathcal{B}}(x_i-\bar{x}_{\mathrm{II}})(e_i-\bar{e}_{\mathrm{II}}) \\
&= O_P(n_{\mathrm{II}}^{-1/2}),
\end{aligned}
$$

and therefore $n_{\mathrm{II}}^{1/2}(\bar{x}_{\mathrm{II}}-\bar{x}_{\mathrm{I}})^{\mathrm{T}}(\hat{\beta}_{\mathrm{II}}-\beta_0) = O_P(1)O_P(n_{\mathrm{II}}^{-1/2}) = O_P(n_{\mathrm{II}}^{-1/2}) = o_P(1)$. We then express (11) as

$$
n_{\mathrm{II}}^{1/2}(\bar{y}_{\mathrm{II,reg}}-\bar{y}_0) = n_{\mathrm{II}}^{1/2}(\bar{e}_{\mathrm{II}}-\bar{e}_{\mathrm{I}}) + n_{\mathrm{II}}^{1/2}(\bar{y}_{\mathrm{I}}-\bar{y}_0) + o_P(1). \qquad \text{(S14)}
$$

Under TPRS in Definition 1, based on (S14), the design variance $\mathrm{var}\{n_{\mathrm{II}}^{1/2}(\bar{y}_{\mathrm{II,reg}}-\bar{y}_0) \mid \mathcal{F}\}$ is approximately

$$
n_{\mathrm{II}}\mathrm{var}(\bar{y}_{\mathrm{I}}-\bar{y}_0 \mid \mathcal{F}) + n_{\mathrm{II}}E\{\mathrm{var}(\bar{e}_{\mathrm{II}} \mid \mathcal{A},\mathcal{F}) \mid \mathcal{F}\} = n_{\mathrm{II}}\left(\frac{1}{n_{\mathrm{I}}}-\frac{1}{N}\right)V_{yy,0} + E\left\{\left(1-\frac{n_{\mathrm{II}}}{n_{\mathrm{I}}}\right)V_{ee,\mathrm{I}} \mid \mathcal{F}\right\}
$$

$$
\begin{aligned}
&= n_{\mathrm{II}}\left(\frac{1}{n_{\mathrm{I}}}-\frac{1}{N}\right)V_{yy,0}+\left(1-\frac{n_{\mathrm{II}}}{n_{\mathrm{I}}}\right)V_{ee,0} \\
&\to f_{\mathrm{II,I}}\,(1-f_{\mathrm{I},0})\,\sigma_{yy}+(1-f_{\mathrm{II,I}})\,\sigma_{ee},\ \text{a.s.},
\end{aligned}
$$

where the second equality follows by Lemma S3.

Under TPRS in Definition 1, $n_{\mathrm{II}}^{1/2}(\bar{y}_{\mathrm{II,reg}}-\bar{y}_0)$ is equivalent to $n_{\mathrm{II}}^{1/2}(\bar{y}_{\mathrm{II,reg}}-\bar{y}_0)\mid(Q_{\mathrm{I}}<\gamma^2)$ without rejective sampling. Similar to (S12), we can show that $\bar{e}_{\mathrm{II}}-\bar{e}_{\mathrm{I}}$ and Q_{I} are asymptotically independent. Moreover, we can show that $\bar{y}_{\mathrm{I}}-\bar{y}_0$ and Q_{I} are asymptotically independent. Based on (S14), $n_{\mathrm{II}}^{1/2}(\bar{y}_{\mathrm{II,reg}}-\bar{y}_0)$ and Q_{I} are asymptotically independent. As a result, $n_{\mathrm{II}}^{1/2}(\bar{y}_{\mathrm{II,reg}}-\bar{y}_0)$ has the same distribution under two-phase sampling with and without rejective sampling.

S3.4 Proof of Proposition 1

We follow the proofs in Li and Ding (2018). Under TPRS in Definition 1, $\bar{u}_{\mathrm{II}}\to E(u)$ a.s., and

$$
\begin{aligned}
\hat{V}_{uv} &= \frac{1}{n_{\mathrm{II}}-1}\sum_{i\in\mathcal{B}}(u_i-\bar{u}_{\mathrm{II}})(v_i-\bar{v}_{\mathrm{II}}) \\
&= \frac{1}{n_{\mathrm{II}}-1}\sum_{i\in\mathcal{B}}\{u_i-E(u)\}\{v_i-E(v)\}-\frac{n_{\mathrm{II}}}{n_{\mathrm{II}}-1}\{\bar{u}_{\mathrm{II}}-E(u)\}\{\bar{v}_{\mathrm{II}}-E(v)\} \\
&\to V_{uv},\ \text{a.s.},
\end{aligned}
$$

where the last line follows from Assumption 1. Next, we have

$$
\begin{aligned}
E\left\{(\hat{V}_{uv}-V_{uv})^2\right\} &= \left(\frac{n_{\mathrm{II}}}{n_{\mathrm{II}}-1}\right)^2\operatorname{var}\left[\frac{1}{n_{\mathrm{II}}}\sum_{i\in\mathcal{B}}\{u_i-E(u)\}\{v_i-E(v)\}-\{\bar{u}_{\mathrm{II}}-E(u)\}\{\bar{v}_{\mathrm{II}}-E(v)\}\right] \\
&= \left(\frac{n_{\mathrm{II}}}{n_{\mathrm{II}}-1}\right)^2\operatorname{var}\left[\frac{1}{n_{\mathrm{II}}}\sum_{i\in\mathcal{B}}\{u_i-E(u)\}\{v_i-E(v)\}\right] \\
&\quad+\operatorname{var}\left[\{\bar{u}_{\mathrm{II}}-E(u)\}\{\bar{v}_{\mathrm{II}}-E(v)\}\right]. \qquad \text{(S15)}
\end{aligned}
$$

To further evaluate (S15), we have

$$
\begin{aligned}
\operatorname{var}\left[\frac{1}{n_{\mathrm{II}}}\sum_{i\in\mathcal{B}}\{u_i-E(u)\}\{v_i-E(v)\}\right] &= \left(\frac{1}{n_{\mathrm{II}}}\right)^2E\left(\sum_{i\in\mathcal{B}}\left[\{u_i-E(u)\}\{v_i-E(v)\}\right.\right. \\
&\quad\left.\left.-\frac{1}{n_{\mathrm{II}}}\sum_{j\in\mathcal{B}}\{u_j-E(u)\}\{v_j-E(v)\}\right]^2\right) \\
&\le \left(\frac{1}{n_{\mathrm{II}}}\right)^2E\left[\sum_{i\in\mathcal{B}}\{u_i-E(u)\}^2\{v_i-E(v)\}^2\right] \\
&\le \frac{1}{n_{\mathrm{II}}}E\left[\frac{1}{n_{\mathrm{II}}}\sum_{i\in\mathcal{B}}\{u_i-E(u)\}^4+\frac{1}{n_{\mathrm{II}}}\sum_{i\in\mathcal{B}}\{v_i-E(v)\}^4\right]
\end{aligned}
$$

$$= \frac{1}{n_{\rm II}} E\left[\{u - E(u)\}^4\right] + \frac{1}{n_{\rm II}} E\left[\{v - E(v)\}^4\right]$$

and

$$\begin{aligned} \mathrm{var}\left[\{\bar{u}_{\rm II} - E(u)\}\{\bar{v}_{\rm II} - E(v)\}\right] &\leq E\left[\{\bar{u}_{\rm II} - E(u)\}^2\{\bar{v}_{\rm II} - E(v)\}^2\right] \\ &\leq E\left[\{\bar{u}_{\rm II} - E(u)\}^4 + \{\bar{v}_{\rm II} - E(v)\}^4\right] \\ &= \frac{1}{n_{\rm II}} E\left[\{u - E(u)\}^4\right] + \frac{1}{n_{\rm II}} E\left[\{v - E(v)\}^4\right]. \end{aligned}$$

Therefore, under Assumption 1,

$$E\left\{(\hat{V}_{uv} - V_{uv})^2\right\} \leq \frac{2}{n_{\rm II}} E\left[\{u - E(u)\}^4\right] + \frac{2}{n_{\rm II}} E\left[\{v - E(v)\}^4\right] \to 0. \tag{S16}$$

By the law of total probability, we have

$$\begin{aligned} E\left\{(\hat{V}_{uv} - V_{uv})^2\right\} &= E\left\{(\hat{V}_{uv} - V_{uv})^2 \mid Q_{\rm I} < \gamma^2\right\} P(Q_{\rm I} < \gamma^2) \\ &\quad + E\left\{(\hat{V}_{uv} - V_{uv})^2 \mid Q_{\rm I} \geq \gamma^2\right\} P(Q_{\rm I} \geq \gamma^2) \\ &\geq E\left\{(\hat{V}_{uv} - V_{uv})^2 \mid Q_{\rm I} < \gamma^2\right\} P(Q_{\rm I} < \gamma^2). \end{aligned}$$

Combining the above inequality with (S16), we obtain

$$E\left\{(\hat{V}_{uv} - V_{uv})^2 \mid Q_{\rm I} < \gamma^2\right\} \leq \{P(Q_{\rm I} < \gamma^2)\}^{-1} E\left\{(\hat{V}_{uv} - V_{uv})^2\right\} = o(1).$$

Therefore, $\hat{V}_{uv} = V_{uv} + o_P(1)$, which completes the proof.

S3.5 Proof of Lemma 2

By the constructions of phase I and II samples and estimators, we have

$$E(T_1 \mid \mathcal{A}, \mathcal{F}) = 0, \; E(T_2 \mid \mathcal{A}, \mathcal{F}) = 0, \; E(T_3 \mid \mathcal{F}) = 0, \tag{S17}$$

and therefore $E(T_k \mid \mathcal{F}) = 0$, for $k = 1, 2, 3$.

We then show the asymptotic variance formulas. First, the asymptotic design variance of T_1 given $\mathcal{F}$ is

$$\begin{aligned} \mathrm{a.var}(T_1 \mid \mathcal{F}) &\equiv \lim_{N\to\infty} \mathrm{var}(T_1 \mid \mathcal{F}) = \lim_{N\to\infty} \left[\mathrm{var}\left\{E(T_1 \mid \mathcal{A}, \mathcal{F}) \mid \mathcal{F}\right\} + E\left\{\mathrm{var}(T_1 \mid \mathcal{A}, \mathcal{F}) \mid \mathcal{F}\right\}\right] \\ &= \lim_{N\to\infty} \left[0 + E\left\{\frac{n_{\rm II}}{N^2} \sum_{i\in\mathcal{A}} \sum_{j\in\mathcal{A}} \frac{\pi_{{\rm II}ij|\mathcal{A}} - \pi_{{\rm II}i|\mathcal{A}}\pi_{{\rm II}j|\mathcal{A}}}{\pi_i^* \pi_j^*} (y_i - \bar{y}_0)(y_j - \bar{y}_0) \mid \mathcal{F}\right\}\right] = V_1, \text{ a.s.}. \end{aligned}$$

Second, the asymptotic design variance of T_2 given $\mathcal{F}$ is

$$\text{a.var}(T_2 \mid \mathcal{F}) \equiv \lim_{N\to\infty} \text{var}(T_2 \mid \mathcal{F}) = \lim_{N\to\infty} \left[\text{var}\left\{E(T_2 \mid \mathcal{A}, \mathcal{F}) \mid \mathcal{F}\right\} + E\left\{\text{var}(T_2 \mid \mathcal{A}, \mathcal{F}) \mid \mathcal{F}\right\}\right]$$

$$= \lim_{N\to\infty} \left[0 + E\left\{\frac{n_{\text{II}}}{N^2} \sum_{i\in\mathcal{A}} \sum_{j\in\mathcal{A}} \frac{\pi_{\text{II}ij|\mathcal{A}} - \pi_{\text{II}i|\mathcal{A}}\pi_{\text{II}j|\mathcal{A}}}{\pi_i^*\pi_j^*}(e_i - \bar{e}_0)(e_j - \bar{e}_0) \mid \mathcal{F}\right\}\right] = V_2, \text{ a.s..}$$

Third, the asymptotic design variance of T_3 given $\mathcal{F}$ is

$$\text{a.var}(T_3 \mid \mathcal{F}) \equiv \lim_{N\to\infty} \text{var}(T_3 \mid \mathcal{F})$$

$$= \lim_{N\to\infty} \left\{\frac{n_{\text{II}}}{N^2} \sum_{i=1}^{N} \sum_{j=1}^{N} \frac{\pi_{\text{I}ij} - \pi_{\text{I}i}\pi_{\text{I}j}}{\pi_{\text{I}i}\pi_{\text{I}j}}(y_i - \bar{y}_0)(y_j - \bar{y}_0)\right\} = V_3, \text{ a.s..}$$

Note that V_1, V_2 and V_3 are finite, guaranteed by Assumption 2.

Finally, we show that all covariances of cross terms are zero asymptotically. By Assumption 2(vii), $\text{a.cov}(T_1, T_2 \mid \mathcal{F}) = 0$ a.s.. Because conditional on $\mathcal{A}$, T_3 is a constant, we then have

$$\text{a.cov}(T_k, T_3 \mid \mathcal{A}, \mathcal{F}) = 0, \; k = 1, 2. \tag{S18}$$

Combining (S17)–(S18), we have

$$\begin{aligned} \text{a.cov}(T_j, T_k \mid \mathcal{F}) &\equiv \lim_{N\to\infty} \text{cov}(T_j, T_k \mid \mathcal{F}) \\ &= \lim_{N\to\infty} E\{\text{cov}(T_j, T_k \mid \mathcal{A}, \mathcal{F}) \mid \mathcal{F}\} \\ &\quad + \lim_{N\to\infty} \text{cov}\{E(T_j \mid \mathcal{A}, \mathcal{F}), E(T_k \mid \mathcal{A}, \mathcal{F}) \mid \mathcal{F}\} \\ &= 0, \; (j \neq k \in \{1, 2, 3\}), \text{ a.s..} \end{aligned}$$

Combining all results completes the proof.

S3.6 Proof of Theorem 4

To derive the asymptotic design property of the two-phase regression estimator, we use the following decomposition:

$$\begin{aligned} \bar{y}_{\text{II,reg}} - \bar{y}_0 &= \bar{y}_{\text{II}} - \bar{y}_{\text{I}} + (\bar{x}_{\text{I}} - \bar{x}_{\text{II}})^{\text{T}}\beta_0 + (\bar{x}_{\text{I}} - \bar{x}_{\text{II}})^{\text{T}}(\hat{\beta}_{\text{II}} - \beta_0) + \bar{y}_{\text{I}} - \bar{y}_0 \\ &= \bar{e}_{\text{II}} - \bar{e}_{\text{I}} + \bar{y}_{\text{I}} - \bar{y}_0 + O_P(n_{\text{II}}^{-1}), \end{aligned}$$

where the second equality follows by $(\bar{x}_{\text{I}} - \bar{x}_{\text{II}})^{\text{T}}(\hat{\beta}_{\text{II}} - \beta_0) = O_P(n_{\text{II}}^{-1})$ due to $\bar{x}_{\text{I}} - \bar{x}_{\text{II}} = O_P(n_{\text{II}}^{-1/2})$ and $\hat{\beta}_{\text{II}} - \beta_0 = O_P(n_{\text{II}}^{-1/2})$.

Therefore, the asymptotic design variance of $\bar{y}_{\text{II,reg}}$ is

$$\begin{aligned} \text{a.var}\left\{n_{\text{II}}^{1/2}(\bar{y}_{\text{II,reg}} - \bar{y}_0) \mid \mathcal{F}\right\} &\equiv \lim_{N\to\infty} \text{var}\left\{n_{\text{II}}^{1/2}(\bar{y}_{\text{II,reg}} - \bar{y}_0) \mid \mathcal{F}\right\} \\ &= \lim_{N\to\infty} \left[\text{var}(n_{\text{II}}^{1/2}\bar{y}_{\text{I}} \mid \mathcal{F}) + E\left\{\text{var}(n_{\text{II}}^{1/2}\bar{e}_{\text{II}} \mid \mathcal{A}, \mathcal{F}) \mid \mathcal{F}_N\right\}\right] \end{aligned}$$

$$
\begin{aligned}
&= \lim_{N\to\infty}\left[\frac{n_{\mathrm{II}}}{N^2}\sum_{i=1}^{N}\sum_{j=1}^{N}\frac{\pi_{\mathrm{I}ij}-\pi_{\mathrm{I}i}\pi_{\mathrm{I}j}}{\pi_{\mathrm{I}i}\pi_{\mathrm{I}j}}(y_i-\bar{y}_0)(y_j-\bar{y}_0)\right.\\
&\quad\left.+E\left\{\frac{n_{\mathrm{II}}}{N^2}\sum_{i\in\mathcal{A}}\sum_{j\in\mathcal{A}}\frac{\pi_{\mathrm{II}ij|\mathcal{A}}-\pi_{\mathrm{II}i|\mathcal{A}}\pi_{\mathrm{II}j|\mathcal{A}}}{\pi_i^*\pi_j^*}(e_i-\bar{e}_0)(e_j-\bar{e}_0)\mid\mathcal{F}\right\}\right]\\
&= \lim_{N\to\infty}\left\{n_{\mathrm{II}}V_{yy,0}+E\left(n_{\mathrm{II}}V_{ee,\mathrm{I}}\mid\mathcal{F}\right)\right\}.
\end{aligned}
$$

S3.7 Proof of Remark 1

For the phase-I and phase-II sample sizes, we have

$$
\sum_{j=1}^{N} I(j\in\mathcal{A}) = n_{\mathrm{I}}, \tag{S19}
$$

$$
\sum_{j\in\mathcal{A}} I(j\in\mathcal{B}) = n_{\mathrm{II}}. \tag{S20}
$$

By taking the expectation of (S19), we obtain

$$
\sum_{j=1}^{N}\pi_{\mathrm{I}j} = n_{\mathrm{I}}, \tag{S21}
$$

By taking the conditional expectation of (S20) given $\mathcal{A}$, we obtain

$$
\sum_{j\in\mathcal{A}}\pi_{\mathrm{II}j|\mathcal{A}} = n_{\mathrm{II}}. \tag{S22}
$$

Multiplying (S19) by $I(i\in\mathcal{A})$ and multiplying (S20) by $I(i\in\mathcal{B})$, we obtain

$$
\sum_{j=1}^{N} I(j\in\mathcal{A})I(i\in\mathcal{A}) = n_{\mathrm{I}}I(i\in\mathcal{A}), \tag{S23}
$$

$$
\sum_{j\in\mathcal{A}} I(j\in\mathcal{B})I(i\in\mathcal{B}) = n_{\mathrm{II}}I(i\in\mathcal{B}). \tag{S24}
$$

By taking the expectation of (S23), we obtain

$$
\sum_{j=1}^{N}\pi_{\mathrm{I}ij} = n_{\mathrm{I}}\pi_{\mathrm{I}i}, \tag{S25}
$$

By taking the conditional expectation of (S24) given $\mathcal{A}$, we obtain

$$
\sum_{j\in\mathcal{A}}\pi_{\mathrm{II}ij|\mathcal{A}} = n_{\mathrm{II}}\pi_{\mathrm{II}i|\mathcal{A}}. \tag{S26}
$$

We now write

$$
\begin{aligned}
\hat{V}_{2,\mathrm{SYG}} &= -\frac{n_{\mathrm{II}}}{N^2}\sum_{i\in\mathcal{B}}\sum_{j\in\mathcal{B}}\frac{\pi_{\mathrm{II}ij|\mathcal{A}}-\pi_{\mathrm{II}i|\mathcal{A}}\pi_{\mathrm{II}j|\mathcal{A}}}{\pi_{\mathrm{II}ij|\mathcal{A}}}\left(\frac{\hat{e}_i-\hat{e}_{\mathrm{II}}}{\pi^*_{\mathrm{II}i}}\right)^{\otimes 2} \\
&\quad +\frac{n_{\mathrm{II}}}{N^2}\sum_{i\in\mathcal{B}}\sum_{j\in\mathcal{B}}\frac{\pi_{\mathrm{II}ij|\mathcal{A}}-\pi_{\mathrm{II}i|\mathcal{A}}\pi_{\mathrm{II}j|\mathcal{A}}}{\pi_{\mathrm{II}ij|\mathcal{A}}}\left(\frac{\hat{e}_i-\hat{e}_{\mathrm{II}}}{\pi^*_{\mathrm{II}i}}\right)\left(\frac{\hat{e}_j-\hat{e}_{\mathrm{II}}}{\pi^*_{\mathrm{II}j}}\right)^{\mathrm{T}} \\
&= T_{2,\mathrm{SYG}}+\hat{V}_2.
\end{aligned}
$$

Moreover,

$$
\begin{aligned}
E\left(T_{2,\mathrm{SYG}}\mid\mathcal{A},\mathcal{F}\right) &= -\frac{n_{\mathrm{II}}}{N^2}E\left\{\sum_{i\in\mathcal{A}}\sum_{j\in\mathcal{A}}\left(\pi_{\mathrm{II}ij|\mathcal{A}}-\pi_{\mathrm{II}i|\mathcal{A}}\pi_{\mathrm{II}j|\mathcal{A}}\right)\left(\frac{\hat{e}_i-\hat{e}_{\mathrm{II}}}{\pi^*_{\mathrm{II}i}}\right)^{\otimes 2}\mid\mathcal{A},\mathcal{F}\right\} \\
&= -\frac{n_{\mathrm{II}}}{N^2}E\left\{\sum_{i\in\mathcal{A}}\left(\sum_{j\in\mathcal{A}}\pi_{\mathrm{II}ij|\mathcal{A}}\right)\left(\frac{\hat{e}_i-\hat{e}_{\mathrm{II}}}{\pi^*_{\mathrm{II}i}}\right)^{\otimes 2}\mid\mathcal{A},\mathcal{F}\right\} \\
&\quad +\frac{n_{\mathrm{II}}}{N^2}E\left\{\sum_{i\in\mathcal{A}}\pi_{\mathrm{II}i|\mathcal{A}}\left(\sum_{j\in\mathcal{A}}\pi_{\mathrm{II}j|\mathcal{A}}\right)\left(\frac{\hat{e}_i-\hat{e}_{\mathrm{II}}}{\pi^*_{\mathrm{II}i}}\right)^{\otimes 2}\mid\mathcal{A},\mathcal{F}\right\} \\
&= -\frac{n_{\mathrm{II}}}{N^2}E\left\{\sum_{i\in\mathcal{A}}\left(n_{\mathrm{II}}\pi_{\mathrm{II}i|\mathcal{A}}\right)\left(\frac{\hat{e}_i-\hat{e}_{\mathrm{II}}}{\pi^*_{\mathrm{II}i}}\right)^{\otimes 2}\mid\mathcal{A},\mathcal{F}\right\} \qquad \text{(S27)} \\
&\quad +\frac{n_{\mathrm{II}}}{N^2}E\left\{\sum_{i\in\mathcal{A}}\pi_{\mathrm{II}i|\mathcal{A}}n_{\mathrm{II}}\left(\frac{\hat{e}_i-\hat{e}_{\mathrm{II}}}{\pi^*_{\mathrm{II}i}}\right)^{\otimes 2}\mid\mathcal{A},\mathcal{F}\right\} \qquad \text{(S28)} \\
&= 0,
\end{aligned}
$$

where (S27) follows by (S26), and (S28) follows by (S22). Therefore, $\hat{V}_{2,\mathrm{SYG}}$ is asymptotically equivalent to $\hat{V}_2$ adding a mean zero term.

We also write

$$
\begin{aligned}
\hat{V}_{3,\mathrm{SYG}} &= -\frac{n_{\mathrm{II}}}{N^2}\sum_{i\in\mathcal{B}}\sum_{j\in\mathcal{B}}\left(\frac{\pi_{\mathrm{I}ij}-\pi_{\mathrm{I}i}\pi_{\mathrm{I}j}}{\pi_{\mathrm{I}ij}\pi_{\mathrm{II}ij|\mathcal{A}}}\right)\left(\frac{y_i-\bar{y}_{\mathrm{II}}}{\pi_{\mathrm{I}i}}\right)^{\otimes 2} \\
&\quad +\frac{n_{\mathrm{II}}}{N^2}\sum_{i\in\mathcal{B}}\sum_{j\in\mathcal{B}}\left(\frac{\pi_{\mathrm{I}ij}-\pi_{\mathrm{I}i}\pi_{\mathrm{I}j}}{\pi_{\mathrm{I}ij}\pi_{\mathrm{II}ij|\mathcal{A}}}\right)\left(\frac{y_i-\bar{y}_{\mathrm{II}}}{\pi_{\mathrm{I}i}}\right)\left(\frac{y_j-\bar{y}_{\mathrm{II}}}{\pi_{\mathrm{I}j}}\right)^{\mathrm{T}} \\
&= T_{3,\mathrm{SYG}}+\hat{V}_3.
\end{aligned}
$$

Moreover,

$$
E(T_{3,\mathrm{SYG}}) = -\frac{n_{\mathrm{II}}}{N^2}E\left\{\sum_{i=1}^N\left(\sum_{j=1}^N\pi_{\mathrm{I}ij}\right)\left(\frac{y_i-\bar{y}_{\mathrm{II}}}{\pi_{\mathrm{I}i}}\right)^{\otimes 2}-\left(\sum_{j=1}^N\pi_{\mathrm{I}j}\right)\sum_{i=1}^N\pi_{\mathrm{I}i}\left(\frac{y_i-\bar{y}_{\mathrm{II}}}{\pi_{\mathrm{I}i}}\right)^{\otimes 2}\right\}
$$

$$= -\frac{n_{\rm II}}{N^2} E\left\{ \sum_{i=1}^N \left(\sum_{j=1}^N \pi_{{\rm I}ij} \right) \left(\frac{y_i - \bar{y}_{\rm II}}{\pi_{{\rm I}i}} \right)^{\otimes 2} - \left(\sum_{j=1}^N \pi_{{\rm I}j} \right) \sum_{i=1}^N \pi_{{\rm I}i} \left(\frac{y_i - \bar{y}_{\rm II}}{\pi_{{\rm I}i}} \right)^{\otimes 2} \right\}$$

$$= -\frac{n_{\rm II}}{N^2} E\left\{ \sum_{i=1}^N n_{\rm I}\pi_{{\rm I}i} \left(\frac{y_i - \bar{y}_{\rm II}}{\pi_{{\rm I}i}} \right)^{\otimes 2} - n_{\rm I} \sum_{i=1}^N \pi_{{\rm I}i} \left(\frac{y_i - \bar{y}_{\rm II}}{\pi_{{\rm I}i}} \right)^{\otimes 2} \right\} = 0, \qquad \text{(S29)}$$

where (S29) follows by (S21) and (S25). Therefore, $\hat{V}_{3,\rm SYG}$ is asymptotically equivalent to $\hat{V}_3$ adding a mean zero term.

S3.8 Proof Theorem S7

We use the following decomposition:

$$\begin{aligned} n_{\rm III}^{1/2}(\bar{y}_{\rm III} - \bar{y}_0) &= n_{\rm III}^{1/2}(\bar{y}_{\rm III} - \bar{y}_{\rm II}) + n_{\rm III}^{1/2}(\bar{y}_{\rm II} - \bar{y}_{\rm I}) + n_{\rm III}^{1/2}(\bar{y}_{\rm I} - \bar{y}_0) \\ &= n_{\rm III}^{1/2}(\bar{c}_{\rm III} - \bar{c}_{\rm II})\beta_{yc,0} + n_{\rm III}^{1/2}\{\bar{y}_{\rm III} - \bar{y}_{\rm II} - (\bar{c}_{\rm III} - \bar{c}_{\rm II})\beta_{yc,0}\} \\ &\quad + n_{\rm III}^{1/2}(\bar{x}_{\rm II} - \bar{x}_{\rm I})\beta_{yx,0} + n_{\rm III}^{1/2}\{\bar{y}_{\rm II} - \bar{y}_{\rm I} - (\bar{x}_{\rm II} - \bar{x}_{\rm I})\beta_{yx,0}\} \\ &\quad + n_{\rm III}^{1/2}(\bar{y}_{\rm I} - \bar{y}_0) \\ &= n_{\rm III}^{1/2}(\bar{c}_{\rm III} - \bar{c}_{\rm II})\beta_{yc,0} + n_{\rm III}^{1/2}(\bar{e}_{yc,\rm III} - \bar{e}_{yc,\rm II}) \\ &\quad + n_{\rm III}^{1/2}(\bar{x}_{\rm II} - \bar{x}_{\rm I})\beta_{yx,0} + n_{\rm III}^{1/2}(\bar{e}_{yx,\rm II} - \bar{e}_{yx,\rm I}) \\ &\quad + n_{\rm III}^{1/2}(\bar{y}_{\rm I} - \bar{y}_0). \end{aligned}$$

We have $D_{x,\rm I} = V_{xx,\rm I}^{-1/2}(\bar{x}_{\rm II} - \bar{x}_{\rm I}) \to \mathcal{N}(0, I_{p_1})$ and $D_{c,\rm II} = V_{cc,\rm II}^{-1/2}(\bar{c}_{\rm III} - \bar{c}_{\rm II}) \to \mathcal{N}(0, I_{p_1+p_2})$. Therefore, we have

$$\begin{aligned} & n_{\rm III}^{1/2}(\bar{y}_{\rm III} - \bar{y}_0) \mid (Q_{x,\rm I} < \gamma_1^2, Q_{c,\rm II} < \gamma_2^2) \\ = \; & n_{\rm III}^{1/2}(\bar{c}_{\rm III} - \bar{c}_{\rm II})\beta_{yc,0} \mid (Q_{x,\rm I} < \gamma_1^2, Q_{c,\rm II} < \gamma_2^2) + n_{\rm III}^{1/2}(\bar{e}_{yc,\rm III} - \bar{e}_{yc,\rm II}) \\ & + n_{\rm III}^{1/2}(\bar{x}_{\rm II} - \bar{x}_{\rm I})\beta_{yx,0} \mid (Q_{x,\rm I} < \gamma_1^2, Q_{c,\rm II} < \gamma_2^2) + n_{\rm III}^{1/2}(\bar{e}_{yx,\rm II} - \bar{e}_{yx,\rm I}) \\ & + n_{\rm III}^{1/2}(\bar{y}_{\rm I} - \bar{y}_0) \\ = \; & n_{\rm III}^{1/2}(\bar{c}_{\rm III} - \bar{c}_{\rm II})\beta_{yc,0} \mid (Q_{c,\rm II} < \gamma_2^2) + n_{\rm III}^{1/2}(\bar{e}_{yc,\rm III} - \bar{e}_{yc,\rm II}) \\ & + n_{\rm III}^{1/2}(\bar{x}_{\rm II} - \bar{x}_{\rm I})\beta_{yx,0} \mid (Q_{x,\rm I} < \gamma_1^2) + n_{\rm III}^{1/2}(\bar{e}_{yx,\rm II} - \bar{e}_{yx,\rm I}) \\ & + n_{\rm III}^{1/2}(\bar{y}_{\rm I} - \bar{y}_0) \\ \to \; & V_{\rm III,1}^{1/2} D_{c,\rm II} \mid \left(D_{c,\rm II}^{\rm T} D_{c,\rm II} < \gamma_2^2\right) + V_{\rm III,2}^{1/2} Z_1 + V_{\rm III,3}^{1/2} D_{x,\rm I} \mid \left(D_{x,\rm I}^{\rm T} D_{x,\rm I} < \gamma_1^2\right) + V_{\rm III,4}^{1/2} Z_2 + V_{\rm III,5}^{1/2} Z_3 \\ \to \; & V_{\rm III,1}^{1/2} L_{p_1+p_2,\gamma_2^2} + V_{\rm III,2}^{1/2} Z_1 + V_{\rm III,3}^{1/2} L_{p_1,\gamma_1^2} + V_{\rm III,4}^{1/2} Z_2 + V_{\rm III,5}^{1/2} Z_3. \end{aligned}$$

S3.9 Proof of Theorem S8

To derive the asymptotic design properties of the three-phase regression estimator, we use the following facts:

$$E(\bar{y}_{\rm III,reg} \mid \mathcal{B}, \mathcal{A}, \mathcal{F}) = \bar{y}_{\rm II,reg} + O_P(n_{\rm III}^{-1}),$$

where the probability distribution in O_P is due to phase-III random sampling given $(\mathcal{B}, \mathcal{A}, \mathcal{F})$, $\bar{y}_{\mathrm{II,reg}} = \bar{y}_{\mathrm{II}} + (\bar{x}_{\mathrm{I}} - \bar{x}_{\mathrm{II}})^{\mathrm{T}} \hat{\beta}_{yx,\mathrm{II}}$, and $\hat{\beta}_{yx,\mathrm{II}}$ is defined as (S6) with z and x being y and x, and

$$E\left(\bar{y}_{\mathrm{II,reg}} \mid \mathcal{A}, \mathcal{F}\right) = \bar{y}_{\mathrm{I}} + O_P(n_{\mathrm{II}}^{-1}),$$

the probability distribution in O_P is due to phase-II random sampling given $(\mathcal{A}, \mathcal{F})$. Therefore, we use the following decomposition:

$$\begin{aligned} \bar{y}_{\mathrm{III,reg}} - \bar{y}_0 &= \bar{y}_{\mathrm{III,reg}} - \bar{y}_{\mathrm{II,reg}} + \bar{y}_{\mathrm{II,reg}} - \bar{y}_{\mathrm{I}} + \bar{y}_{\mathrm{I}} - \bar{y}_0 \\ &= \left\{ \bar{y}_{\mathrm{III}} + \begin{pmatrix} \bar{x}_{\mathrm{I}} - \bar{x}_{\mathrm{III}} \\ -\bar{a}_{\mathrm{III}} \end{pmatrix}^{\mathrm{T}} \hat{\beta}_{yc,\mathrm{III}} \right\} - \left\{ \bar{y}_{\mathrm{II}} + (\bar{x}_{\mathrm{I}} - \bar{x}_{\mathrm{II}})^{\mathrm{T}} \hat{\beta}_{yx,\mathrm{II}} \right\} \\ &\quad + \left\{ \bar{y}_{\mathrm{II}} - \bar{y}_{\mathrm{I}} + (\bar{x}_{\mathrm{I}} - \bar{x}_{\mathrm{II}})^{\mathrm{T}} \hat{\beta}_{yx,\mathrm{II}} \right\} + \bar{y}_{\mathrm{I}} - \bar{y}_0 \\ &= (\bar{e}_{yc,\mathrm{III}} - \bar{e}_{yc,\mathrm{II}}) + (\bar{e}_{yx,\mathrm{II}} - \bar{e}_{yx,\mathrm{I}}) + \bar{y}_{\mathrm{I}} - \bar{y}_0 + O_P(n_{\mathrm{III}}^{-1}). \end{aligned}$$

By repeated application of conditional expectation arguments, the asymptotic design variance of $\bar{y}_{\mathrm{III,reg}}$ is

$$\mathrm{a.var}\left\{ n_{\mathrm{III}}^{1/2} (\bar{y}_{\mathrm{III,reg}} - \bar{y}_0) \mid \mathcal{F} \right\} = \lim_{N \to \infty} \left\{ n_{\mathrm{III}} V_{yy,0} + E\left(n_{\mathrm{III}} V_{e_{yx} e_{yx},\mathrm{I}} \mid \mathcal{F}\right) + E\left(n_{\mathrm{III}} V_{e_{yc} e_{yc},\mathrm{II}} \mid \mathcal{F}\right) \right\}.$$

Then, under Assumptions 2 and S1, we establish the result in Theorem S8.

S3.10 Proof of Theorem 5

We first state the regularity conditions for Theorem 5.

Assumption S2 *The following conditions hold for the population parameter ξ_0 and the population estimating function $\bar{s}_0(\cdot)$:*

(i) The population parameter ξ_0 lies within a closed interval $\mathcal{I}_\xi$.

(ii) The function $s_i(\cdot)$ is bounded.

(iii) The population estimating function $\bar{s}_0(\xi)$ converges uniformly to $s_0(\xi)$ on $\mathcal{I}_\xi$ as $N \to \infty$, and the equation $s_0(\xi) = 0$ has a unique root within the interior of $\mathcal{I}_\xi$.

(iv) The limiting function $s_0(\xi)$ is strictly increasing in each component of ξ and absolutely continuous, with finite first and second derivatives in $\mathcal{I}_\xi$. Additionally, the derivative $\partial s_0(\xi)/\partial \xi$ is bounded away from zero within $\mathcal{I}_\xi$.

(v) The following conditions on the population quantities are satisfied:

$$\sup_{\xi \in \mathcal{I}_\xi} N^{1/2} |\bar{s}_0(\xi_0 + N^{-1/2}\xi) - \bar{s}_0(\xi_0) - s_0(\xi_0 + N^{-1/2}\xi) - s_0(\xi_0)| \to 0,$$

and

$$\sup_{\xi \in \mathcal{I}_\xi} N^{-1} \sum_{i=1}^{N} |s_i(\xi_0 + N^{-1/2}\xi) - s_i(\xi_0)| = O_P(N^{-1/2}).$$

Assumption S2(i)–(iv) are common for M-estimators (Serfling, 1980). Assumption S2(v) applies to differentiable estimating functions and has been examined by Wang and Opsomer (2011) for non-differentiable estimating functions. Wang and Opsomer (2011) demonstrate that under appropriate conditions on the probability mechanism generating the y_i values and the function $s(y_i;\xi)$, Assumption S2(v) holds with probability one.

We will now show the results in Theorem 5. Under Assumption S2, and using the theory for M-estimators for differentiable estimating functions or the results of Wang and Opsomer (2011) for non-differentiable estimating functions, we can express

$$\bar{s}_{\mathrm{II}}(\bar{\xi}_{\mathrm{II}}) - \bar{s}_0(\xi_0) = \bar{s}_{\mathrm{II}}(\xi_0) - \bar{s}_0(\xi_0) + \Gamma_s^{\mathrm{T}}(\bar{\xi}_{\mathrm{II}} - \xi_0) + o_P(n_{\mathrm{II}}^{-1/2}). \tag{S30}$$

By Assumption S2(iv), $\bar{s}_0(\xi)$ is smooth, implying $\bar{s}_0(\xi_0) = O_P(N^{-1})$, $\bar{s}_{\mathrm{II}}(\bar{\xi}_{\mathrm{II}}) = O_P(n_{\mathrm{II}}^{-1})$, and the left-hand side of (S30) is $o_P(n_{\mathrm{II}}^{-1/2})$.

Because Assumption 2 holds for $s_i = s_i(\xi_0)$, we define the residual vector as

$$e_i^s = s_i - B_0^{\mathrm{T}} x_i,$$

where

$$B_0 = \left\{\sum_{i=1}^{N} (x_i - \bar{x}_0)^{\otimes 2}\right\}^{-1} \sum_{i=1}^{N} (x_i - \bar{x}_0)\{s_i(\xi_0) - \bar{s}_0(\xi_0)\}^{\mathrm{T}}.$$

Under TPRS in Definition 2, we have

$$n_{\mathrm{II}}^{1/2}\{\bar{s}_{\mathrm{II}}(\xi_0) - \bar{s}_0(\xi_0)\} \mid \left(Q_{\mathrm{I}} < \gamma^2\right) \to (V_1^s)^{1/2} L_{p,\gamma^2} + (V_2^s)^{1/2} Z_1 + (V_3^s)^{1/2} Z_2, \tag{S31}$$

where Z_1 and Z_2 are standard normal variables, and $(L_{p,\gamma^2}, Z_1, Z_2)$ are jointly independent.

Combining the results (S30) and (S31), we obtain

$$n_{\mathrm{II}}^{1/2}(\bar{\xi}_{\mathrm{II}} - \xi_0) \mid \left(Q_{\mathrm{I}} < \gamma^2\right) \to \Gamma_s^{\mathrm{T}}(V_1^s)^{1/2} L_{p,\gamma^2} + \Gamma_s^{\mathrm{T}}(V_2^s)^{1/2} Z_1 + \Gamma_s^{\mathrm{T}}(V_3^s)^{1/2} Z_2,$$

as $n_{\mathrm{II}} \to \infty$.
